# Chemical Fingerprinting of Synthetic Polymers via Direct Insertion Probe Mass Spectrometry

**DOI:** 10.1021/acs.macromol.5c03190

**Published:** 2026-03-03

**Authors:** Ville H. Nissinen, Nea Heilala, Krista Grönlund, Paavo Auvinen, Mika Suvanto, Jarkko J. Saarinen, Janne Jänis

**Affiliations:** Department of Chemistry and Sustainable Technology, University of Eastern Finland, P. O. Box 111, FI-80101 Joensuu, Finland

## Abstract

We report on the chemical fingerprinting of synthetic polymers using direct insertion probe mass spectrometry (DIP-MS), an analytical approach requiring only minimal sample preparation. A total of 38 different polymers were analyzed using temperature-programmed DIP-MS with atmospheric pressure chemical ionization (APCI) to establish a comprehensive spectral library. The studied polymers included homo- and copolymers from various classes, such as polyolefins, polyethers, polyesters, polyamides, styrenics, thermoplastic elastomers, and fluoropolymers. DIP-APCI-MS provided detailed structural information, enabling reliable identification of nearly all polymers based on their characteristic thermal decomposition patterns. Moreover, the utilization of temperature-programmed approach allowed monitoring of sample degradation as a function of temperature, further aiding polymer identification. Overall, temperature-programmed DIP-APCI-MS proved to be a robust and efficient method for the chemical fingerprinting of synthetic polymers, with potential applications in areas such as microplastic analysis and plastic recycling.

## Graphical abstract

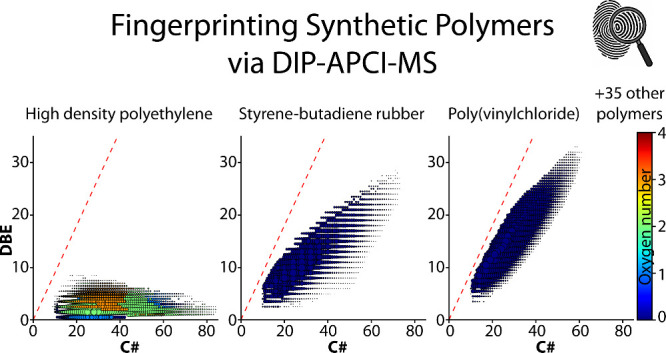

## Introduction

Over the past few decades, mass spectrometry (MS) has arguably become one of the most versatile chemical analysis techniques. Advances in instrumentation, ionization techniques and sample introduction methods have rendered mass spectrometry suitable for a wide variety of analytes, ranging from small molecules to large macromolecules.^1,2^ Particularly, the use of various “soft” ionization techniques, causing little or no fragmentation of the analytes, has enabled the effective analysis of polymeric materials.^3,4^ The most common “soft” ionization methods include electrospray ionization (ESI), matrix assisted laser desorption/ionization (MALDI) and atmospheric pressure chemical ionization (APCI).^3^ These techniques have proven especially useful when combined with high-resolution instruments for the analysis of complex mixtures. Consequently, mass spectrometry has found widespread applications in fields such as metabolomics, petroleomics and analysis of biopolymers such as proteins.^1^

Despite its powerful analytical capabilities, mass spectrometry remains somewhat underutilized in the field of synthetic polymer analysis. Conventionally, mass spectrometric analysis of synthetic polymers has been dominated by MALDI and ESI techniques, enabling analysis of intact polymer structures.^4^ In the case of ESI, the analytes must be introduced into the ion source in liquid state, which limits its applicability to certain synthetic polymers with limited solubility in ESI compatible solvents.^3,4^ In MALDI, the sample of interest is mixed with a matrix, typically an aromatic acid in suitable solvent, although solvent-free sample preparation techniques have also emerged.^5,6^ Upon laser irradiation, analytes are transferred into gas-phase ions with the assistance of the matrix that absorbs energy and facilitates their desorption and ionization.^7,8^ Therefore, sample preparation, including the selection of matrix and solvents, is a critical step for successful MALDI analysis.^6^ The optimal choice of matrix depends on the polymer to be analyzed, and identifying a suitable matrix for nonpolar analytes can be challenging due to their low ionization efficiency.^6,9^ Consequently, certain polymer types, such as polyolefins and thermosets, cannot be effectively analyzed with either MALDI or ESI.^10^ Furthermore, both ESI and MALDI are prone to ion suppression/charge competition effects, potentially leading to biased analyte response.^3,11^

To overcome the limitations, destructive mass spectrometric strategies offer a viable alternative.^10^ These methods rely on the controlled degradation of polymer samples to generate low molar mass species that retain chemical and architectural information and can be effectively analyzed using conventional ionization techniques.^10^ The sample degradation can be achieved using chemical methods or reactive ionization, e.g., reactive surface-assisted laser desorption/ionization (SALDI)^10,12^ However, the most common methods are based on controlled thermal decomposition of the sample.^10^

Pyrolysis gas chromatography coupled with mass spectrometry (Py-GC-MS) stands out as the most established analytical pyrolysis method, and it has been widely applied to characterization of synthetic polymers.^13–15^ In this technique, the sample of interest is subjected to high temperatures typically under an inert atmosphere. The resulting volatiles and thermal degradation products are then chromatographically separated, followed by characterization via mass spectrometry.^16^ Py-GC-MS analysis often requires only minimal sample preparation, but the duty cycles are typically relatively long due to the chromatographic separation involved.^16,17^ Another limitation is that analytes incompatible with gas chromatography (e.g., those with high molar mass or highly polar) cannot be effectively analyzed by Py-GC-MS, although derivatization can partially mitigate the issue.^13,17^ Consequently, only relatively small polymer fragments are typically detected, which may lead to a loss of structural information and possibly limit the applicability of Py-GC-MS, for example, in the characterization of complex copolymers.^14,17^

Recently, there has been growing interest in chromatography-free mass spectrometric methods that enable streamlined analysis of solid samples with little or no sample preparation. Among the most utilized chromatography-free techniques are direct analysis in real time (DART), direct insertion probe (DIP) and atmospheric pressure solids analysis probe (ASAP).^3,10,12^ All three methods rely on controlled heating of the sample inside the ion source. ASAP utilizes a hot stream of nitrogen to volatilize and/or thermally decompose the sample within an APCI source.^3,10^ In the case of DIP, the sample heating is realized using a dedicated heating element, offering more precise temperature control than ASAP.^10,12^ Furthermore, the DIP accessory is compatible with both APCI and atmospheric pressure photoionization (APPI) sources, ensuring efficient ionization of a broad range of analytes.^18^ DART, on the other hand, uses a stream of hot metastable gas to volatilize and ionize analytes.^3,10^ The sample on interest is either placed directly to the hot gas stream or the sample can be preheated separately, with the resulting volatiles directed into the metastable gas flow for ionization.^19^

The chromatography-free mass spectrometric approaches have found applications in areas such as analysis of environmental contaminants and forensic science.^20–22^ Furthermore, these methods have garnered attention also in the context of plastic characterization. Many of the previous studies have focused on organic plastic additive identification,^23–31^ but also studies on synthetic polymer characterization have been published.^19,29,32–44^ The primary advantage of DIP, ASAP and DART over conventional polymer composition analysis techniques (e.g., chromatographic and spectroscopic methods) is the elimination of extensive sample preparation steps.^3^ A distinctive feature of the direct mass spectrometric approaches is the controlled chain degradation under mild conditions, typically resulting in the formation of relatively large polymeric fragments that contain relevant structural information.^10^ Moreover, rapid ionization and detection of the desorbed compounds and thermal degradation products offers shorter measurement times compared to chromatographic methods, and minimizes the risk of secondary reactions, thereby preserving the integrity of the analytes.

Previously, we have demonstrated temperature-programmed DIP-MS analysis of plastic additives, particularly brominated flame retardants.^45–47^ The utilized approach was also found to provide detailed information on polymer composition.^45^ In the present study, we focused particularly on the structural characterization and chemical fingerprinting of a wide variety of synthetic polymers using temperature-programmed DIP-APCI-MS. While DART, ASAP, and DIP have been used to study a few polymer grades, no single technique has yet been applied to a wide range of polymers. Especially, the analysis of copolymers with these techniques has been sparsely reported. In this work, synthetic polymers from various classes were analyzed to evaluate method’s applicability across different polymer types and to establish a comprehensive library of their spectral fingerprints.

## Experimental Section

### Materials and General Considerations

Details of the studied polymers, along with their abbreviations, are presented in Table 1. All thermoplastic polymers were analyzed as received. Polydimethylsiloxane (PDMS) sample was prepared using Sylgard 184 two-part elastomer kit according to the manufacturer’s instructions. Shortly, the base material and curing agent were thoroughly mixed in a mass ratio of 10:1. After degassing in vacuum, the mixture was cured for 4 h at 65 °C. DIP-MS consumables, Hirschmann melting point tubes and Pallflex Tissuquartz 2500QAT-UP quartz filters, were prebaked at 500 °C in ambient air for at least 5 h to remove any volatile impurities.

**Table 1:** Polymers Used in the Study

**polymer**	**Abbr.**	**supplier**	**grade/remarks**
**polyolefins**			
high density polyethylene	HDPE	Borealis Polymers	Nakiku CG 8410
low density polyethylene	LDPE	Borealis Polymers	Nakiku CA 7230
liner low density polyethylene	LLDPE	Sigma-Aldrich	melt index 100.00 g/10 min at 190 °C/2.16 kg
polypropylene	PP	Borealis Polymers	HD120MO
ethylene-octene copolymer	EOC	Dow Chemical Company	Engage 8400
cyclic olefin polymer	COP	Zeon Chemical	Zeonex 330R
cyclic olefin copolymer	COC	Ticona	Topas 5013
**vinyl polymers**			
polystyrene	PS	Sigma-Aldrich	*M*_n_ ∼ 170,000; *M*_w_ ∼ 350,000
poly(vinylchloride)	PVC	Sigma-Aldrich	*M*_n_ ∼ 47,000; *M*_w_ ∼ 80,000
polybutadiene	PB	Sigma-Aldrich	*M*_n_ ∼ 5000
poly(vinyl alcohol)	PVA	Sigma-Aldrich	*M*_w_ 31,000–50,000
poly(methyl methacrylate)	PMMA	Röhm	Plexiglas 7N
poly(vinylpyrrolidone)	PVP	Fluka	K 25; *M*_w_ ∼ 25,000
poly(4-vinylpyridine)	P4VP	Sigma-Aldrich	*M*_w_ ∼ 60,000
**polyesters and amides**			
poly(lactic acid)	PLA	Sigma-Aldrich	*M*_n_ 40,000
poly(ethylene terephthalate)	PET	Eastman	Eastapak 9921
poly(butylene terephthalate)	PBT	Celanese	Celanex 2404 MT Natural
polycarbonate	PC	Covestro	Makrolon 2858, bisphenol A-based
polyamide 6	PA6	EMS-Grivory	Grilon BS 23
polyamide 6,6	PA66	Sigma-Aldrich	Nylon 6,6
**polyethers and amines**			
polyoxymethylene	POM	DuPont	Delrin PC652 NC010
polyoxymethylene copolymer	co-POM	Celanese	Hostaform MT8U01 Natural
poly(tetrahydrofuran)	PTHF	Sigma-Aldrich	*M*_n_ ∼ 2900
poly(ethylene glycol)	PEG	Sigma-Aldrich	*M*_n_ ∼ 2050
polyethylenimine	PEI	Sigma-Aldrich	*M*_n_ ∼ 10,000; *M*_w_ ∼ 25,000; branched structure
**styrene copolymers**			
high impact polystyrene	HIPS	INEOS Styrolution	Empera 622N
styrene-butadiene rubber	SBR	Ravelast Polymers	
styrene-acrylonitrile	SAN	BASF	LURAN 358N
acrylonitrile-butadiene-styrene	ABS	Sabic	Cycolac MG47F
styrene-isoprene-styrene	SIS	Kraton Corporation	Kraton D
styrene-butadiene-styrene	SBS	INEOS Styrolution	Styrolux 656C
methyl methacrylate-acrylonitrile-butadiene-styrene	MABS	INEOS Styrolution	Terlux 2812 TR
**miscellaneous**			
thermoplastic polyurethane elastomer	TPU	Noveon	ESTANE 58881, polyether type
thermoplastic polyurethane elastomer	TPU	Dow Chemical Company	Isoplast ETP
thermoplastic polyester elastomer	TPC-ET	DuPont	Hytrel 4056
polytetrafluoroethylene	PTFE	Guarniflon	G400
ethylene-tetrafluoroethylene-hexafluoropropylene	EFEP	Daikin Chemicals	NEOFLON RP-4020
polydimethylsiloxane	PDMS	Dow Chemical Company	Sylgard 184

### Mass Spectrometry

All mass spectra were recorded using a high-resolution Bruker timsTOF (Bruker Daltonics GmbH, Bremen, Germany) quadrupole time-of-flight instrument. The mass spectrometer was equipped with a combination of APCI source and a DIP accessory. A small piece of polymer was placed on a prebaked glass capillary, which was capped with a quartz filter and placed inside the ion source set to a temperature of 150 °C. The APCI vaporizer temperature was then increased from 150 to 450 °C with 50 °C steps within 10.5 min (Figure 1). All measurements were conducted in a positive ion mode and the most important MS parameters were as follows: a capillary voltage of 4000 V, a corona current of 4000 nA, a nebulizer pressure of 2.0 bar, a dry gas flow of 5.0 L/min and a dry gas temperature of 200 °C. Ion transfer parameters (a multipole RF of 250 V_pp_, a collision RF of 600 V_pp_, a transfer time of 140 μs, and a prepulse storage of 12 μs) were adjusted to enable efficient detection of ions in a *m*/*z* range of 150–1000. The mass spectrometer was externally calibrated with a polystyrene standard and controlled with Bruker otofControl 6.2 software.

**Figure 1:**
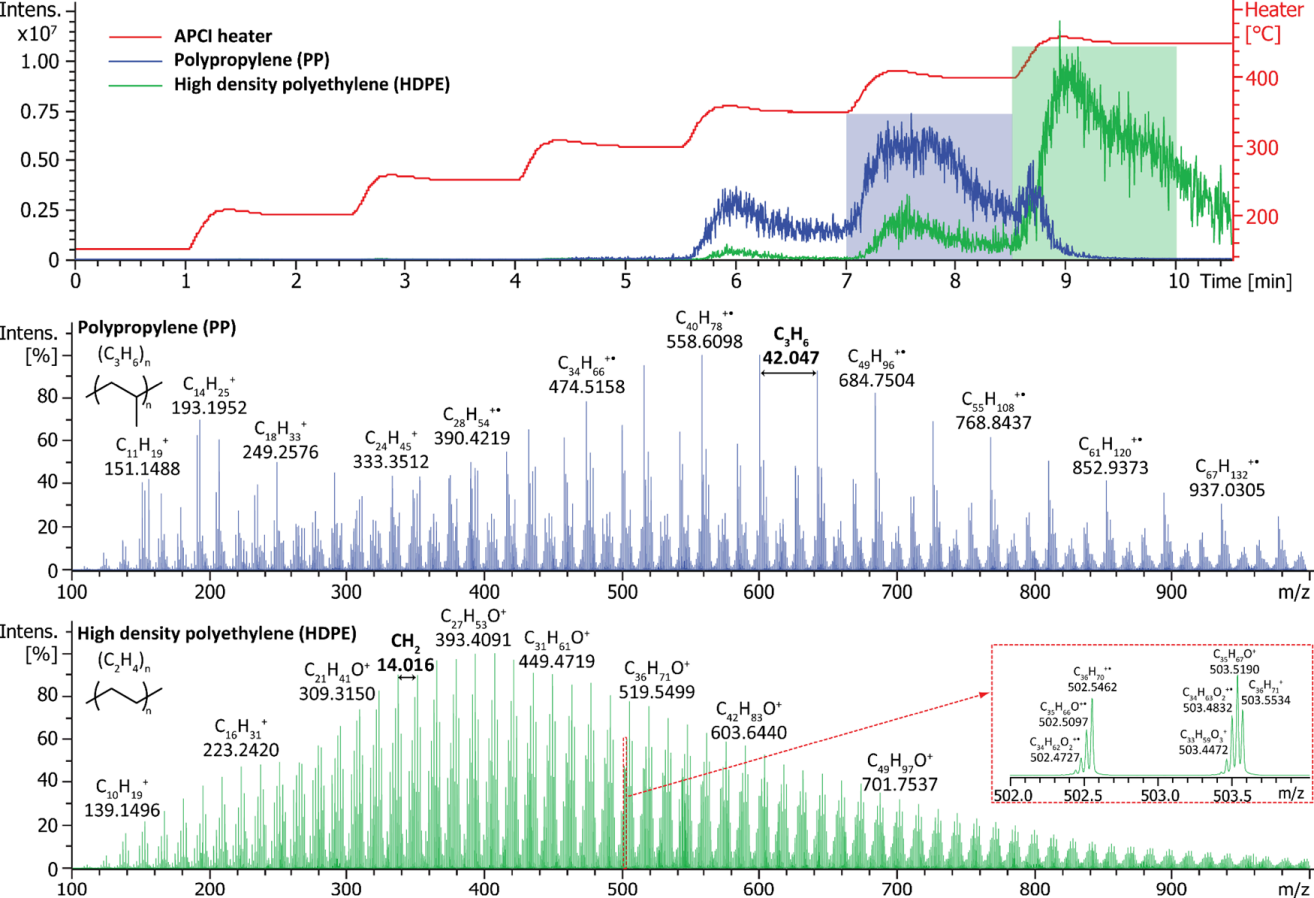
DIP-MS analysis of PP (blue traces) and HDPE (green traces) samples. The top panel shows total ion chromatograms (TICs) obtained using a vaporizer temperature program from 150 to 450 °C (red trace), whereas the middle and bottom panels present averaged mass spectra obtained for polypropylene and polyethylene at 400 and 450 °C, respectively.

### Data Analysis and Visualization

Bruker DataAnalysis 5.1 software was utilized in the analysis of the acquired MS data. Each data set was internally recalibrated using custom-made reference mass lists for improved mass accuracy. Part of the data was treated semimanually, while for more complex spectra, the SmartFormula tool was used for the assignment of ion formulas. Elemental boundaries and other constraints were adjusted individually for each polymer. Visualization of the MS data, including the preparation of double bond equivalent (DBE) versus carbon number plots and Kendrick mass defect (KMD) plots, was accomplished using the PyC2MC software by Sueur and co-workers.^48^

## Results and Discussion

The following sections describe the DIP-APCI-MS characteristics of synthetic homo- and copolymers from various classes. For the majority of the polymers analyzed, once the main degradation region was reached, the thermal decomposition patterns remained highly consistent over the subsequent temperature steps. For these materials, only the spectrum corresponding to the main degradation temperature is presented, whereas multiple spectra are shown for polymers that exhibited clear temperature-dependent changes in the thermal degradation products. In addition to thermal polymer fragment ions, DIP-MS analysis of several industrial-grade samples yielded ions assigned to organic plastic additives, mainly antioxidants. These additive signals were observed primarily in the spectra recorded at 300–350 °C. However, these low-temperature spectra are not emphasized, as the primary objective of this study was to investigate polymer specific degradation behavior and mass-spectral fingerprints rather than the embedded additive packages. In the context of this work, additive signals cannot be reliably leveraged for polymer identification, and for the majority of the analyzed polymer grades, their presence is not expected to notably influence the reproducibility or interpretability of the DIP-MS fingerprints. The analysis of organic plastic additives by temperature-programmed DIP-APCI-MS has been described in detail in our previous publications.^45–47^

### Analysis of Polyolefins

Figure 1 presents the temperature-programmed DIP-MS analysis of two common polyolefins, namely polypropylene and high density polyethylene. Both polymer grades generated a plethora of thermal fragments in the utilized measurement conditions. For polypropylene, multiple series of hydrocarbon species separated by 42.047 Da (C_3_H_6_) were observed, indicating that thermal fragmentation of PP preferably occurs at the position of tertiary carbon atoms. The observed ions were mostly radical cations with DBE values ranging from 2 to 5. For HDPE exhibiting an unbranched structure, an even more complex spectrum was obtained. The thermal fragmentation products consisted of multiple ion series separated by 14.016 Da (CH_2_). In addition to pure hydrocarbons, oxygenated species containing up to three oxygen atoms were observed. The formation of oxygenated hydrocarbon fragments was observed also in the analysis of PP, but to a significantly lesser extent than in the case of HDPE analysis. The formation of oxygenated species in DIP-APCI-MS measurement of polyolefins has been previously reported and attributed to side reactions occurring inside the ion source, likely due to the presence of residual oxygen and water.^32^

Based on the total ion chromatograms (TICs) obtained in the temperature-programmed DIP-MS analysis of PP and HDPE samples (Figure 1 top panel), the degradation of PP and HDPE occurred mainly at 400 and 450 °C, respectively. The obtained decomposition temperatures are in accordance with a previous thermogravimetric study.^49^ Hence, the use of a temperature-programmed approach allowed monitoring of the sample degradation as a function of temperature, further facilitating the polymer identification.

The analysis of other studied ethylene-based polymers, namely LDPE, LLDPE, and EOC, resulted in similar mass spectra and decomposition temperature profiles as in the case of HDPE as illustrated in Figures S1–S3 in the Supporting Information. Hence, the studied ethylene polymer grades could not be reliably distinguished from each other, although the DIP-APCI-MS analysis of LDPE with a highly branched structure resulted in a more pronounced formation of pure hydrocarbon species compared to the analysis of other studied ethylene polymer grades. It has been previously shown that ion mobility mass spectrometry (IM-MS) holds a potential for the identification of such closely related polymer grades.^32^ Our plan is to pursue this further in our future studies.

Two common cyclic olefin polymer grades namely, COP and COC, were also analyzed using DIP-MS. COP is a homopolymer manufactured via ring-opening metathesis polymerization of norbornene-based cyclic monomers, followed by hydrogenation, whereas COC is prepared via copolymerization of ethene and norbornene.^50^ Both polymers exhibited high thermal stability and decomposition products were primarily detected at 450 °C. In the mass spectra of both COP and COC samples, multiple ion series were observed, as depicted in Figure 2, with the determined mass differences corresponding to the polymer structures. It is noteworthy that oxygenated hydrocarbon species were relatively abundant in the mass spectrum of COP, whereas they were virtually absent in the mass spectrum recorded from the COC sample.

**Figure 2:**
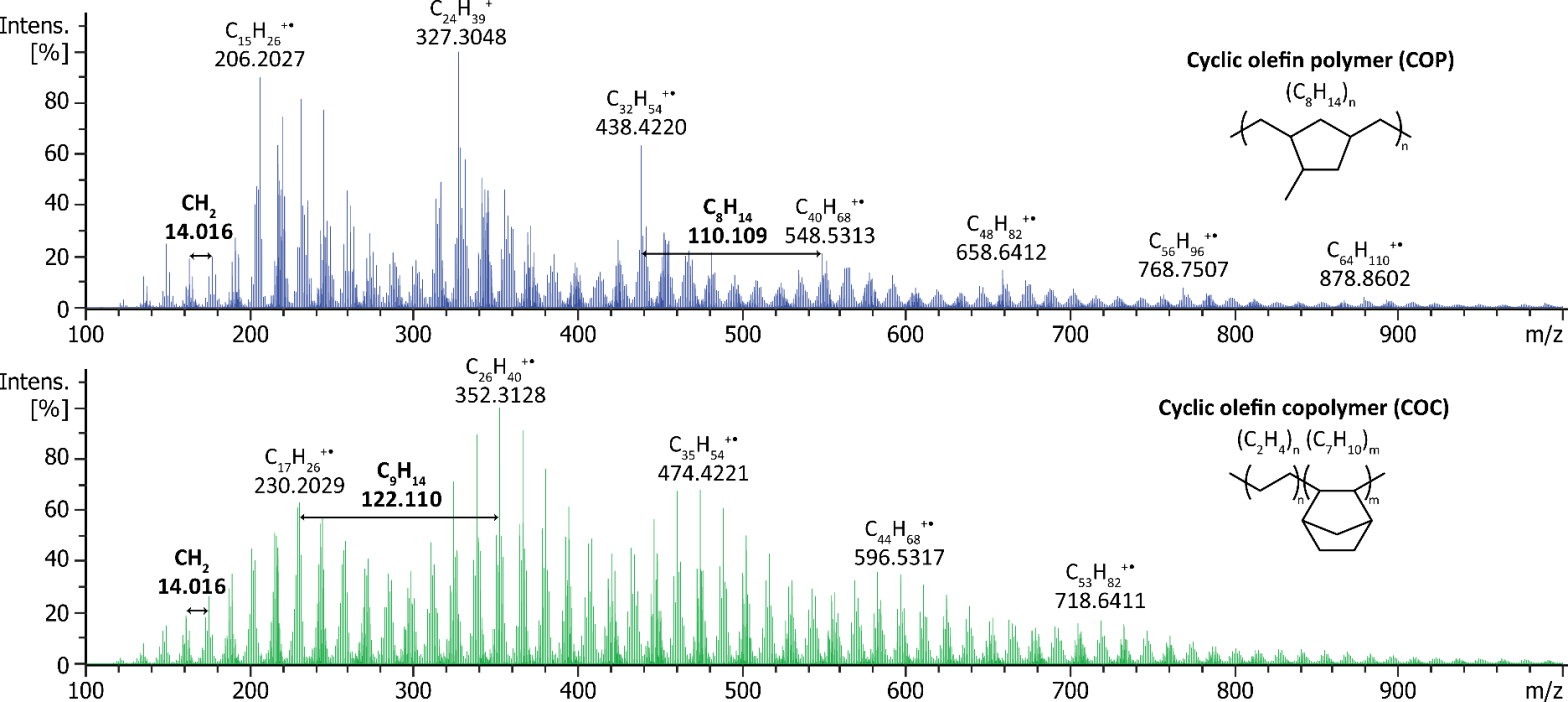
Averaged DIP-MS spectra of COP (top) and COC (bottom) samples obtained at 450 °C.

### Analysis of Other Vinyl Polymers

Other studied vinyl polymers demonstrated varying behavior in the DIP-MS analysis. The analysis of PS, PMMA, PB, PVP, and P4VP resulted in simple spectra, exhibiting signals arising from small thermal polymer fragments, consisting of only a few monomer units (Figures S4–S8). For example, the most abundant ions in the obtained spectra for PS and PMMA were C_16_H_15_^+^ observed at *m*/*z* 207.117 and C_10_H_16_O_4_^+^ observed at *m*/*z* 200.105, respectively. Both base peaks were hence assigned to dimeric fragments derived from the corresponding polymers. On the other hand, DIP-MS analysis of PVA and PVC resulted in notably more complex mass spectra, as depicted in Figures S9 and S10. At high temperatures (450 °C) both PVA and PVC generated a broad distribution of closely separated hydrocarbon ions with DBE values increasing as a function of *m*/*z*. Chlorinated species were totally absent from the DIP-MS spectrum of PVC, while some polymer fragments observed in the spectrum of PVA exhibited a low oxygen content.

The observed variance between different vinyl polymers was attributed to their different decomposition mechanisms under the utilized measurement conditions. For example, PS is known to thermally degrade through radical chain scission of the C–C polymer main chain, ultimately resulting in the formation of styrenic products with alkane, alkene, and dialkene backbones.^51^ PVC, on the other hand, has been reported to decompose via dehydrochlorination, resulting in the formation of HCl and polyenes, which can undergo further reactions.^52^ Similarly to PVC, PVA decomposes via dehydration generating a variety of polyenes and carbonyl compounds.^53,54^ Hence, the results provided by DIP-MS are in concordance with previous literature regarding the decomposition of vinyl polymers.

### Analysis of Polyesters and Polyamides

All analyzed polyesters exhibited relatively simple mass spectra. In accordance with the previous reports, PC was found to be thermally stable with a degradation onset temperature exceeding 400 °C.^55^ The resulting mass spectrum, as shown in Figure S11, exhibited almost exclusively C_14_H_13_O_2_^+^ ions observed at *m*/*z* 213.0912. This ion likely corresponds to demethylated fragment of bisphenol A, one of the two primary monomers of PC. Similar spectral fingerprints have been previously reported for bisphenol A-based synthetic polymers using desorption ionization through hole alumina membrane mass spectrometry (DIUTHAME-MS), which can be considered as a variation of SALDI.^56^

For PLA, multiple series of ions separated by 72.021 Da, consistent with its repeating unit (C_3_H_4_O_2_), were present in the mass spectrum obtained at 400 °C. The most abundant ions were C_18_H_25_O_12_^+^ and C_21_H_29_O_14_^+^, arising from protonated lactic acid hexa- and heptamers (Figure S12). A similar spectral fingerprint for PLA using ASAP-MS has been previously reported.^36^

A characteristic feature of polymers derived from terephthalic acid (PET and PBT) was the presence of C_8_H_5_O_3_^+^ ion observed at *m*/*z* 149.023 in their mass spectra. The signal was assigned to [M + H]^+^ ion of terephthalic anhydride, which is also a common thermal fragmentation product of phthalate acid esters—widely used plasticizers in variety of plastic applications. Therefore, to confirm the presence of PET and PBT in samples, additional depolymerization product ions must be considered. For both PET and PBT, ions corresponding to one and two repeating units, among other degradation products, were observed (Figures S13 and S14), providing a characteristic chemical signature for their identification.

Figure 3 presents DIP-MS spectra of two structurally closely related polyamides, namely polyamide 6 (PA6) and polyamide 6,6 (PA66). Thermal decomposition of both studied polyamides occurred mainly at 450 °C. DIP-MS analysis of PA6 resulted predominantly in the formation of caprolactam oligomers with a general formula (C_6_H_11_NO)_*n*_. In the case of PA66, the monomeric fragment C_12_H_23_N_2_O_2_^+^ was observed among other species. Notably, several of the observed PA66 thermal fragments (e.g., C_18_H_31_N_4_O_2_^+^) can only originate from a structure, in which the orientation of the amide group alternates with each repeating unit. Therefore, these characteristic ions can be used to distinguish PA66 from PA6. The obtained DIP-MS results align well with the previous literature regarding the thermal decomposition of polyamides. In the case of PA6, cyclic oligomers are the primary products.^57^ In contrast, significantly more complex product distribution, including linear and cyclic polymer fragments and monomeric units as well as secondary reaction products, has been observed for PA66.^57^

**Figure 3:**
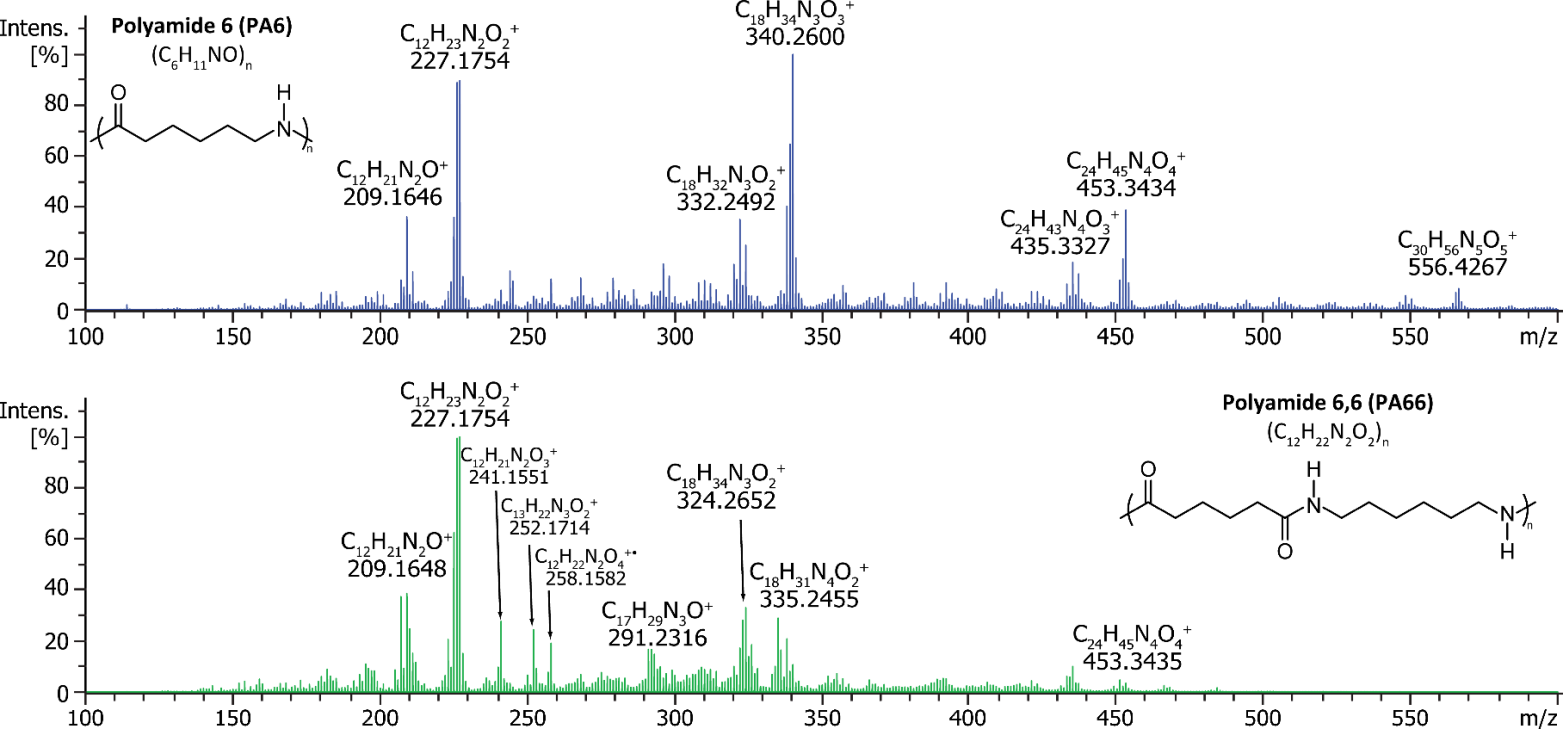
Averaged DIP-MS spectra of PA6 (top) and PA66 (bottom) samples obtained at 450 °C.

Previously, the identification of various polyamide grades using DART-MS has been reported.^19,38,41^ The main observed ions for PA6 and PA66 were identical for both DIP-MS and DART-MS. However, DART-MS analysis yielded simpler spectra, exhibiting almost exclusively signals of oligomeric fragments, likely due to milder ionization conditions employed. Consequently, Abe et al. concluded that MS/MS analysis of the main degradation product (C_12_H_23_N_2_O_2_^+^) was necessary to reliably distinguish between PA6 and PA66 at the utilized ionization conditions.^38^ In contrast, the results presented herein indicated that DIP-MS holds the potential to differentiate between these polyamide grades even without tandem MS. It should be noted, however, that the use of higher DART gas temperature or thermal desorption and pyrolysis DART-MS may likewise enable reliable discrimination between PA6 and 66 without MS/MS. Previous studies have reported speciation analysis of different polyamide grades using DIUTHAME-MS.^56^

### Analysis of Polyethers and Polyamines

DIP-MS spectra of PEG (Figure S15) exhibited multiple ion series separated by 44.026 Da, consistent with its repeating unit (C_2_H_4_O). The observed thermal PEG fragments were predominantly saturated, and only minor amounts of DBE 1 and 2 species were detected. Kendrick mass defect (KMD) analysis has proven to be an effective tool for the visualization of complex mass spectra of polymers.^58–63^ Figure 4 presents a modified KMD plot derived from DIP-MS data of PEG obtained at 400 °C with C_2_H_4_O (44.0262 Da) as the base unit. Each horizontal line in the plot represents a homologous series of ions, the members of which differ solely by the amount of C_2_H_4_O units in their structure. In contrast, ion series with varying KMD values correspond to different end group modifications. Consistent with previous reports, the DIP-MS data indicated that dominant products under mild pyrolysis conditions were dihydroxy-terminated oligomers, having the general formula H_2_O­(C_2_H_4_O)_*n*_.^64^ Additionally, ions corresponding to various end group modifications, including alkyl ether, vinyl ether and aldehyde terminations, were identified. A similar spectra for PEG has been previously reported in a DART-MS study by Cody et al.^19^

**Figure 4:**
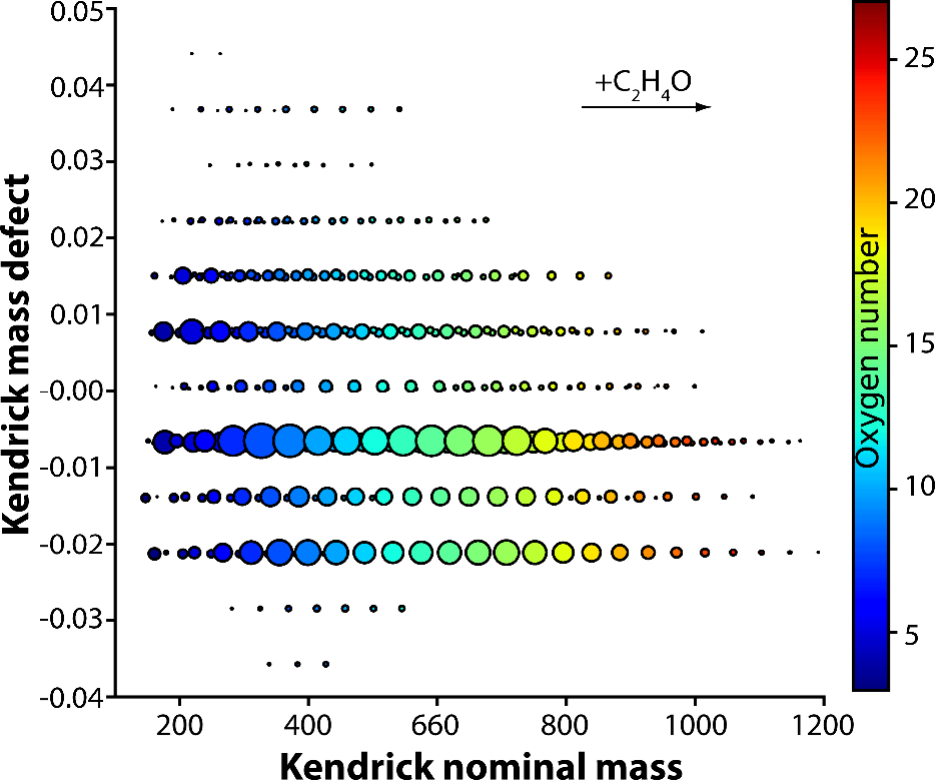
Modified KMD plot derived from DIP-MS data obtained for PEG at 400 °C. C_2_H_4_O (44.0262 Da) was used as the base unit. Size and color of the dots indicate normalized intensity and oxygen number of the ions, respectively.

For PTHF, the base peak of spectrum obtained at 350 °C was observed at *m*/*z* 215.1642 and assigned to C_12_H_23_O_3_^+^ (Figure S16). The signal likely originated from a PTHF trimer bearing dialdehyde end groups. Multiple other PTHF degradation products with a DBE value of 2 were observed in the low *m*/*z* region of the spectra, while saturated PTHF oligomers dominated the higher *m*/*z* region. The most abundant degradation products above *m*/*z* 300 exhibited the general formula [C_4_H_11_O­(C_4_H_8_O)_*n*_]^+^, suggesting protonated PTHF oligomers terminated with butyl ether and hydroxyl end groups. Additionally, H_2_O­(C_4_H_8_O)_*n*_ (dihydroxyl end groups) and C_3_H_8_O­(C_4_H_8_O)_*n*_ (propyl ether and hydroxyl end groups) products were relatively abundant. The results obtained with DIP-MS are consistent with previous reports on the thermal degradation behavior of PTHF under similar conditions.^65^

POM homo- and copolymers were also analyzed using temperature-programmed DIP-MS and the resulting spectra have been presented in Figures S17 and S18. Analysis of both POM grades yielded significantly weaker signals compared to analysis of other studied polyethers. Thermal degradation and pyrolysis of POM has been previously reported to result predominantly in the formation of low molecular weight products, such as formaldehyde, which could not be efficiently detected with the instrumentation used in this study.^16,66^ Nevertheless, a series of larger POM decomposition product ions with formula [C_2_HO_2_(CH_2_O)_*n*_]^+^ was detected, enabling reliable identification of POM by DIP-MS. The mass spectrum of POM homopolymer exhibited also multiple series of ions separated by 14.016 Da (CH_2_), which were found to be characteristic for different polyethylene grades. According to its technical datasheet, the studied POM grade (Delrin PC652 NC010) contains “advanced systems of lubricants”, providing a reasonable explanation for the observed hydrocarbon additive. POM copolymer, the composition of which was not explicitly known, could not be reliably distinguished from the POM homopolymer based on polymer fragment ions alone. However, the presence of characteristic additives provided a spectral fingerprint that enabled their discrimination by DIP-MS.

The results of DIP-MS analysis of branched PEI are presented in Figure S19. The spectra obtained at 350 °C exhibited two main series of ions separated by 43.042 Da, corresponding to its repeating unit (C_2_H_5_N). The main degradation products were acyclic PEI oligomers terminated with amine end groups, NH_3_(C_2_H_5_N)_*n*_, and cyclic or vinyl terminated oligomers with formula (C_2_H_5_N)_*n*_. The results obtained using DIP-MS are in concordance with a previous Py-GC-MS study of branched PEI degradation products by Coralli et al.^67^

### Analysis of Styrene Copolymers

Several styrene copolymers were characterized using DIP-MS. In accordance with the results obtained for general purpose polystyrene (see Figure S4), a distinctive feature of all polymers containing styrene blocks (HIPS, SBS, and SIS) was the presence of small styrene oligomer signals (*n* ≈ 2–5) in the corresponding spectra obtained above 400 °C. Additionally, the spectra of HIPS and SBS recorded at 400 °C (Figure 5 top and middle panels) exhibited signals arising from butadiene oligomers (C_4_H_6_)_*n*_, whereas no oligomers containing both styrene and butadiene were detected. The absence of mixed styrene-butadiene oligomers was attributed to the grafted and block type copolymer structures of HIPS and SBS, respectively.^68^ The polybutadiene fragment signals present in the DIP-MS spectrum of SBS had notably higher relative intensities compared to those of HIPS, which is consistent with the higher typical butadiene content of SBS (60–70 wt %) with respect to HIPS (below 20 wt %).^68–70^ As depicted in Figure S20, SIS demonstrated spectral features similar to those of HIPS and SBS, but with the relatively weak butadiene oligomer signals replaced by abundant polyisoprene fragment ions with the general formula of [(C_5_H_8_)_*n*_]^+•^.

**Figure 5:**
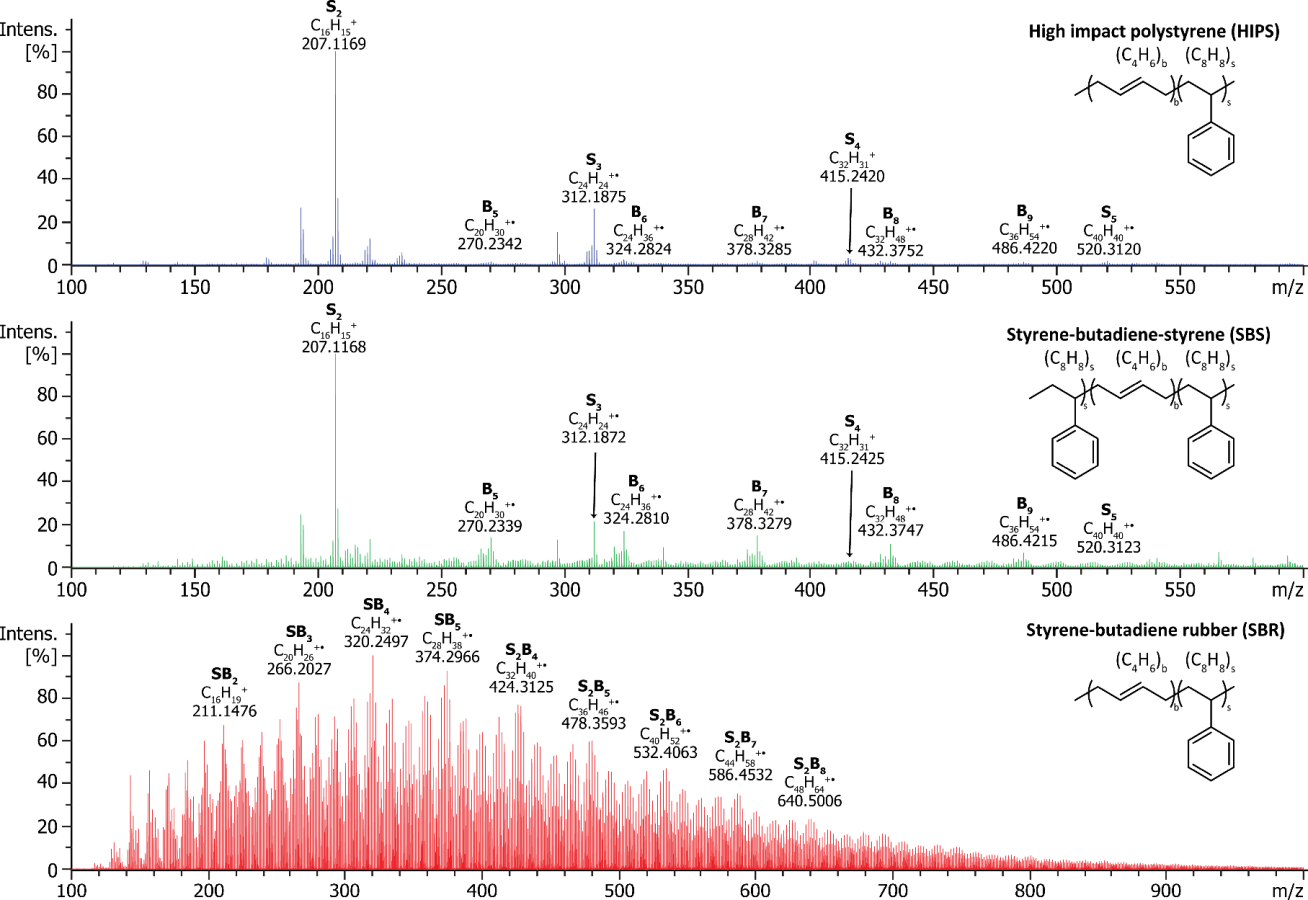
DIP-MS analysis of HIPS (top), SBS (middle), and SBR (bottom). For HIPS and SBS, averaged spectra obtained at 400 °C are presented, whereas the spectrum of SBR was recorded at 450 °C. The observed polymer fragments were designated as S_s_B_b_, where s and b indicate the number of styrene and butadiene monomer units, respectively.

Contrary to HIPS and SBS, the DIP-MS spectrum of SBR recorded at 450 °C exhibited a relatively broad distribution of hydrocarbon signals, with peaks appearing at each nominal mass (Figure 5, bottom panel). The most abundant ions were assigned to oligomers containing one or two styrene monomer units and between two and eight butadiene units. The plethora of hydrocarbon species observed in the DIP-MS analysis is consistent with the random copolymer structure of SBR. Thereby, DIP-MS allowed also for the reliable identification of various copolymers composed of styrene and butadiene.

Several of the studied polymers, e.g., HDPE, PVC and SBR, generated a distribution of closely separated ions in the DIP-MS analysis. Nevertheless, the spectra showed subtle differences that enabled polymer identification. These features are illustrated in Figure 6, presenting DBE versus carbon number plots for each polymer. A distinctive feature of HDPE was the presence of oxygenated species alongside pure hydrocarbons in the DIP-MS spectrum. Additionally, the observed ions displayed relatively low and uniform DBE values across the carbon number range. In contrast, the DBE plot for PVC revealed abundant ions in the vicinity of the planar aromatic limit, indicative of the high degree of unsaturation. For SBR, the DBE values generally increased as a function of carbon number, but not as steeply as for PVC, which could be rationalized by the presence of butadiene units in the observed SBR fragments.

**Figure 6:**
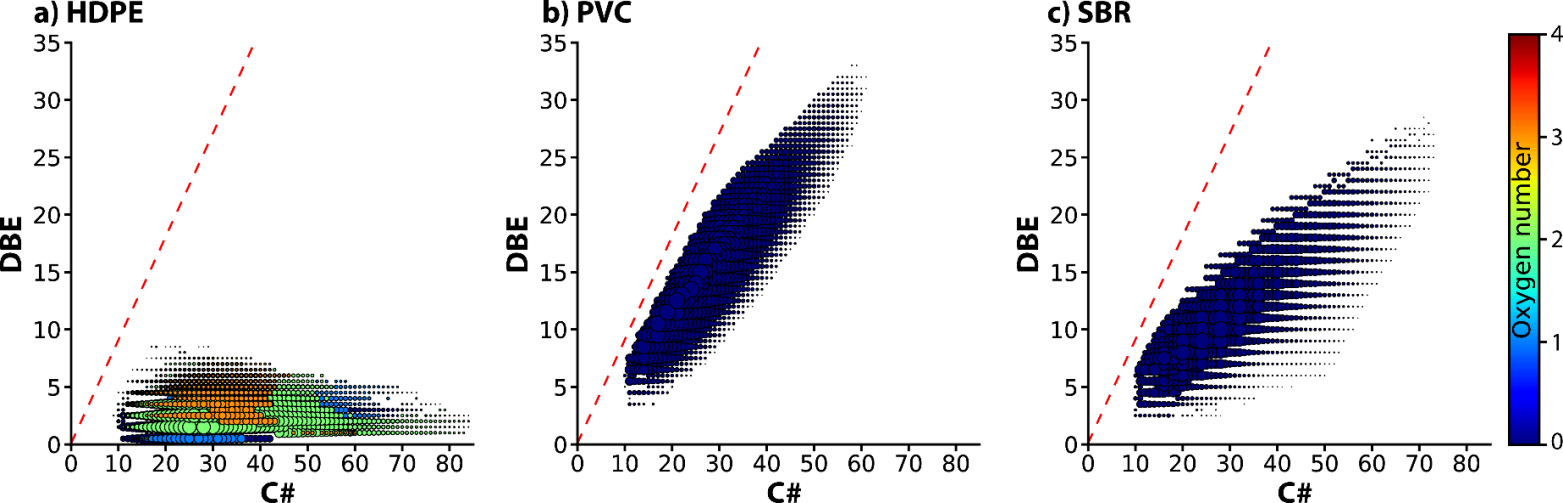
DBE vs carbon number distributions derived from DIP-MS analysis of (a) HDPE, (b) PVC, and (c) SBR. For each polymer, the DBE distributions were determined from the averaged mass spectrum obtained at 450 °C. Size and color of the dots indicate normalized intensity and oxygen number of the ions, respectively. The red dashed lines represent the planar aromatic limit (DBE ≈ 0.9 × C#).

The DIP MS spectrum of SAN revealed numerous polymer fragment signals corresponding to various styrene-acrylonitrile oligomers with the general formula (C_8_H_8_)_*n*_(C_3_H_3_N)_*m*_, as depicted in Figure S21. The observed SAN decomposition products were consistent with its statistical random copolymer structure.^68^ In addition to SAN fragments, DIP-MS analysis of ABS yielded butadiene oligomers, which were primarily detected at 400 °C as illustrated in Figure S22. The formation of polybutadiene fragments reflects the presence of isolated polybutadiene domains in the polymer structure, a pattern also observed for HIPS. Indeed, ABS exhibits a polymer architecture similar to that of HIPS, in which polybutadiene particles are dispersed within a SAN matrix.^68^ Furthermore, MABS could be distinguished from ABS by the presence of PMMA dimer signal (C_10_H_16_O_4_^+•^ at *m*/*z* 200.104) in its spectrum, verifying the incorporation of methyl methacrylate units (Figure S23). Overall, DIP-MS proved capable of providing detailed compositional information on styrene copolymers, enabling their reliable identification.

### Analysis of Other Polymer Grades

For PTFE, no polymer fragment ions were observed in the DIP-MS analysis. The high thermal stability of PTFE likely prevented its characterization with this type of mild pyrolysis technique. Previously, the chemical fingerprint of PTFE, comprising a series of (CF_2_)_*n*_ peaks, was successfully obtained using thermal desorption and pyrolysis DART-MS, which is capable of reaching temperatures up to 600 °C.^19^ Hence, the highest operating temperature of 470 °C of the used APCI source is a minor limitation of the DIP-MS method in the context of polymer analysis. Characterization of a less thermally stable partially fluorinated copolymer EFEP was, nevertheless, possible and the obtained mass spectrum is presented in Figure S24. KMD analysis has also been demonstrated to be useful in the visualization of MS data of fluoropolymers.^34^ Figure 7 presents a modified KMD plot derived from DIP-MS data of EFEP obtained at 450 °C, using CF_2_ (49.9968 Da) as the repeating unit. The KMD analysis revealed several series of ions with identical KMD values, as represented by horizontal lines in the plot. These ions differ solely by the amount of CF_2_ repeating units (or their multiples such as C_2_F_4_ or C_3_F_6_), indicating a homologous series. Vertically aligned ions, on the contrary, correspond to different end group modifications of ions with otherwise identical composition. The diagonal patterns in the plot reflect ions separated by the amount of CH_2_ units in their structure, corresponding to structural variations arising from the incorporation of ethylene units. Hence, the observed polymer fragments aligned with the terpolymer structure of EFEP containing ethylene, tetrafluoroethylene and hexafluoropropylene units.^71^

**Figure 7:**
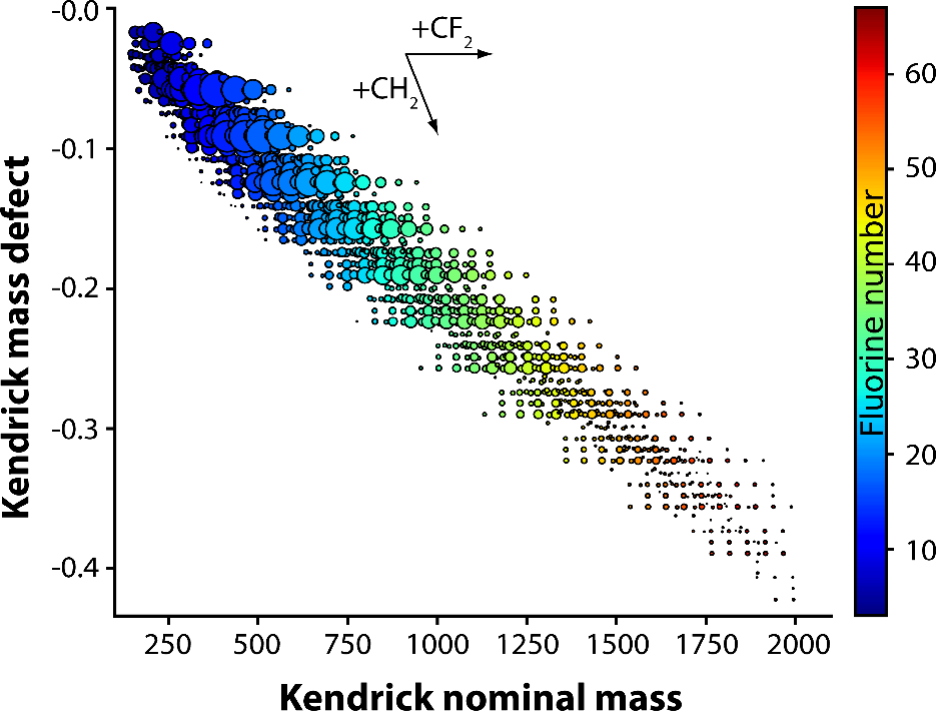
Modified KMD plot derived from DIP-MS data obtained for EFEP at 450 °C. CF_2_ (49.9968 Da) was used as the base unit. Size and color of the dots indicate normalized intensity and fluorine number of the ions, respectively.

The DIP-MS analysis of PDMS yielded a relatively simple mass spectrum, exhibiting mainly two series of ions, each separated by 74.019 Da (Figure S25). The signals corresponded to ions with the general formula [H­(C_2_H_6_OSi)_*n*_]^+^ (*n* ≈ 3–7) and their demethylated analogues, which exhibited the highest abundances. The thermal degradation products of PDMS observed in this study are consistent with previous direct probe and ASAP-MS studies on PDMS-containing copolymers, which suggested that the small PDMS oligomers possess cyclic structures.^33,35^

Also, several engineering thermoplastic elastomers were analyzed by DIP-MS. A distinctive spectral feature of both studied TPU grades (Estane 5881 and Isoplast) was the presence signals arising from methylene diphenyl diisocyanate (MDI) (C_15_H_10_N_2_O_2_^+•^ at *m*/*z* 250.074 and C_14_H_12_N_2_O^+•^ at *m*/*z* 224.094), a principal component of the hard polyurethane segments of aromatic TPUs. Additionally, the spectrum of Estane 5881 TPU exhibited characteristics spectral features of PTHF, consistent with its polyether type soft segments (Figure S26). For the other studied TPU grade (Isoplast), a relatively abundant ion C_21_H_24_N_2_O_4_^+•^ (*m*/*z* 368.1732) was observed, suggesting the presence of butanediol chain extender bound to MDI (Figure S27). However, no abundant ions reflecting the structure of the soft segments were observed. It is noteworthy that the structure of Isoplast TPU has not been defined in detail in its technical documentation. In general, the results of this study are in accordance with the previous literature regarding the structural characterization of TPUs using direct mass spectrometric techniques, although direct comparison is challenging due to variations in the structures of the copolymers analyzed.^29,33,35^ DIP-MS analysis of the Hytrel 4056 thermoplastic polyester elastomer presented in Figure S28 revealed characteristic features of both PBT and PTHF. Therefore, the obtained mass spectrum is consistent with the reported copolymer structure of Hytrel-brand elastomers, which comprise crystalline PBT hard segments and amorphous polyether soft segments.^72^

## Conclusions

DIP-APCI-MS was found to provide detailed structural information on a vast variety of synthetic polymer grades. Each studied polymer, except for PTFE and certain POM and PE grades, exhibited a unique fingerprint spectrum composed of characteristic thermal fragment ions, enabling their identification. These spectral features have been summarized in Table 2. Moreover, the temperature-programmed nature of the measurements introduced a degree of temporal separation between the analytes and simultaneously enabled monitoring of the sample degradation as a function of temperature, further facilitating polymer identification. As a degradative approach, DIP-MS inherently circumvents the isotope-distribution broadening that can complicate MS analysis of intact high molar mass polymers by MALDI or ESI,^3^ potentially providing cleaner and more interpretable fingerprint spectra. On the other hand, detailed information on end groups and molecular weight distribution is largely lost during the analysis, but this did not hinder reliable identification of the polymers examined here. The utilized analysis approach was found to be especially useful in the compositional characterization of copolymers, enabling distinction of even structurally similar polymers. Additionally, DIP-MS shed further light on the thermal decomposition mechanism of polymers. The established spectral library is easily expandable. Future work should explore the potential of ion mobility mass spectrometry to distinguish between polymers that yielded similar mass spectra, such as different PE grades. Additionally, the applicability of the DIP-MS method for the analysis of polymer mixtures should be evaluated.

**Table 2:** Characteristic Spectral Features for Different Polymer Types Based on Temperature-Programmed DIP-APCI-MS Analysis

**polymer**	**observation temperature (°C)**	**characteristic spectral features**
PE	450	broad distribution of C_*x*_H_*y*_O_0–4_^+^ ions with low DBE values (1–5). The most abundant ions separated by 14.016 Da (CH_2_)
PP	400	broad distribution of C_*x*_H_*y*_O_0–3_^+^ ions with low DBE values (2–5). The most abundant ions separated by 42.047 Da (C_3_H_6_)
COP	450	broad distribution of C_*x*_H_*y*_O_0–2_^+^ ions. The most abundant ions separated by 14.016 Da (CH_2_) or 110.109 Da (C_8_H_14_)
COC	450	broad distribution of C_*x*_H_*y*_^+^ ions. The most abundant ions separated by 14.016 Da (CH_2_) or 122.110 Da (C_9_H_14_)
PS	450	base peak C_16_H_15_^+^. Minor ions C_24_H_24_^+•^, C_32_H_31_^+^, and C_40_H_40_^+•^
PB	400	series of [(C_4_H_6_)_*n*_]^+•^ and [H_2_(C_4_H_6_)_*n*_]^+•^ ions (*n* ≈ 4–20), each separated by 54.047 Da
PMMA	400	base peak C_10_H_16_O_4_^+•^. Series of [C_4_H_5_O(C_5_H_8_O_2_)_*n*_]^+^ ions (*n* ≈ 1–5) separated by 100.052 Da
PVP	450	series of [H(C_6_H_9_NO)_*n*_]^+^ ions (*n* ≈ 2–6) separated by 111.068 Da
P4VP	450	C_14_H_15_N_2_^+^, C_21_H_22_N_3_^+^, C_28_H_28_N_4_^+•^, and C_35_H_35_N_5_^+•^
PVC	450	broad distribution of C_*x*_H_*y*_^+^ ions with DBE values increasing as a function of carbon number
PVA	450	broad distribution of C_*x*_H_*y*_O_0–2_^+^ ions with DBE values increasing as a function of carbon number
PLA	400	series of [H(C_3_H_4_O_2_)_*n*_]^+^ ions (*n* ≈ 3–15) separated by 72.021 Da
PET	450	series of [H(C_10_H_8_O_4_)_*n*_]^+^, [C_8_H_5_O_3_(C_10_H_8_O_4_)_*n*_]^+^, and [C_7_H_5_O(C_10_H_8_O_4_)_*n*_]^+^ ions (*n* ≈ 0–4), each separated by 192.042 Da
PBT	400	series of [(C_12_H_12_O_4_)_*n*_]^+•^, [C_8_H_5_O_3_(C_12_H_12_O_4_)_*n*_]^+^, and [C_12_H_11_O_3_(C_12_H_12_O_4_)_*n*_]^+^ ions (*n* ≈ 0–3), each separated by 220.073 Da. Also other major ions observed
PC	450	base peak C_14_H_13_O_2_^+^. Minor ions C_21_H_17_O_4_^+^, C_32_H_28_O_6_^+•^, and C_48_H_42_O_9_^+•^
PA6	450	series of [H(C_6_H_11_NO)_*n*_]^+^ ions (*n* ≈ 2–5) separated by 113.084 Da
PA66	450	base peak C_12_H_23_N_2_O_2_^+^. Minor ions C_12_H_21_N_2_O^+^, C_18_H_34_N_3_O_2_^+^, C_18_H_31_N_4_O_2_^+^, etc.
PTHF	350	base peak C_12_H_23_O_3_^+^. Series of [C_4_H_11_O(C_4_H_8_O)_*n*_]^+^, [H_3_O(C_4_H_8_O)_*n*_]^+^, and [C_3_H_9_O(C_4_H_8_O)_*n*_]^+^ ions (*n* ≈ 3–10) each separated by 72.058 Da
PEG	400	series of [H_3_O(C_2_H_4_O)_*n*_]^+•^ ions (*n* ≈ 5–25) separated by 44.026 Da. Also other ion series with the same separation detected
POM	350	C_6_H_11_O_5_^+^, C_6_H_11_O_6_^+^, C_7_H_13_O_6_^+^, and a series of [C_2_HO_2_(CH_2_O)_*n*_]^+^ ions (*n* ≈ 5–15) separated by 30.011 Da
PEI	400	series of [NH_4_(C_2_H_5_N)_*n*_]^+^ and [H(C_2_H_5_N)_*n*_]^+^ ions (*n* ≈ 4–15), each separated by 43.042 Da
HIPS	400	styrene and butadiene oligomers. See entries for PS and PB
SBS	400	styrene and butadiene oligomers. See entries for PS and PB
SBR	450	broad distribution of C_*x*_H_*y*_^+^ ions. The most abundant ions exhibit the general formula [(C_8_H_8_)_1–2_(C_4_H_6_)_2–8_]^+•^
SIS	400–450	isoprene oligomer ions [(C_5_H_8_)_*n*_]^+•^ (*n* ≈ 8–16) separated by 68.062 Da observed at 400 °C. Styrene oligomers observed mainly at 450 °C (see entry for PS)
SAN	400	acrylonitrile-styrene ions with the general formula [(C_8_H_8_)_1–4_(C_3_H_3_N)_1–4_]^+•^
ABS	400	acrylonitrile-styrene and butadiene oligomers. See entries for SAN and PB
MABS	400–450	acrylonitrile-styrene, butadiene and methyl methacrylate oligomers. See entries for SAN, PB, and PMMA
TPU	400	ions C_15_H_10_N_2_O_2_^+•^ and C_14_H_12_N_2_O^+•^ indicative for MDI-based TPUs
PTFE	n/a	n/a
EFEP	450	multiple series of C_*x*_H_*y*_F_*z*_^+•^ ions separated by either 28.031 Da (C_2_H_4_) or 49.996 Da (CF_2)_
PDMS	450	series of [CH_3_OSi(C_2_H_6_OSi)_*n*_]^+^ and [H(C_2_H_6_OSi)_*n*_]^+^ ions (*n* ≈ 3–7) each separated by 74.019 Da

Overall, temperature-programmed DIP-MS has proven to enable comprehensive characterization of polymers. When combined with its previously demonstrated ability to analyze plastic additives, even quantitatively, this approach offers a versatile tool for plastic analysis.^45,46^ Beyond fundamental characterization, the method’s ability to rapidly analyze solid samples with minimal preparation also highlights its potential for applications in areas such as microplastic identification, plastic recycling, and quality control of polymer materials.

## Supplementary Material



## Notes

Total ion chromatograms and selected mass spectra from the DIP-MS analysis of polymer grades not presented in the main article (PDF)
