# Supplementary material for: Chemical Fingerprinting of Synthetic Polymers via Direct Insertion Probe Mass Spectrometry
Source: Macromolecules. 2026 Mar 3;59(6):3753–64. doi: 10.1021/acs.macromol.5c03190 (PMC13019992; doi:10.1021/acs.macromol.5c03190)
Supplement: Supplementary file 1 [file ma5c03190_si_001.pdf]

Supporting information

Chemical Fingerprinting of Synthetic Polymers via Direct Insertion Probe Mass Spectrometry

Ville H. Nissinen*, Nea Heilala, Krista Grönlund, Paavo Auvinen, Mika Suvanto, Jarkko J. Saarinen, and Janne Jänis

Department of Chemistry and Sustainable Technology, University of Eastern Finland, P.O. Box 111, FI-80101 Joensuu, Finland

*Corresponding author. Email: ville.nissinen@uef.fi

Contents

Figure S1. DIP-MS analysis of LDPE.

Figure S2. DIP-MS analysis of LLDPE.

Figure S3. DIP-MS analysis of EOC.

Figure S4. DIP-MS analysis of PS.

Figure S5. DIP-MS analysis of PMMA.

Figure S6. DIP-MS analysis of PB.

Figure S7. DIP-MS analysis of PVP.

Figure S8. DIP-MS analysis of P4VP.

Figure S9. DIP-MS analysis of PVA.

Figure S10. DIP-MS analysis of PVC.

Figure S11. DIP-MS analysis of PC.

Figure S12. DIP-MS analysis of PLA.

Figure S13. DIP-MS analysis of PET.

Figure S14. DIP-MS analysis of PBT.

Figure S15. DIP-MS analysis of PEG.

Figure S16. DIP-MS analysis of PTHF.

Figure S17. DIP-MS analysis of POM.

Figure S18. DIP-MS analysis of co-POM.

Figure S19. DIP-MS analysis of PEI.

Figure S20. DIP-MS analysis of SIS.

Figure S21. DIP-MS analysis of SAN.

Figure S22. DIP-MS analysis of ABS.

Figure S23. DIP-MS analysis of MABS.

Figure S24. DIP-MS analysis of EFEP.

Figure S25. DIP-MS analysis of PDMS.

Figure S26. DIP-MS analysis of Estane 58881 polyether type TPU.

Figure S27. DIP-MS analysis of Isoplast TPU.

Figure S28. DIP-MS analysis of Hytrel 4056 TPC-ET.

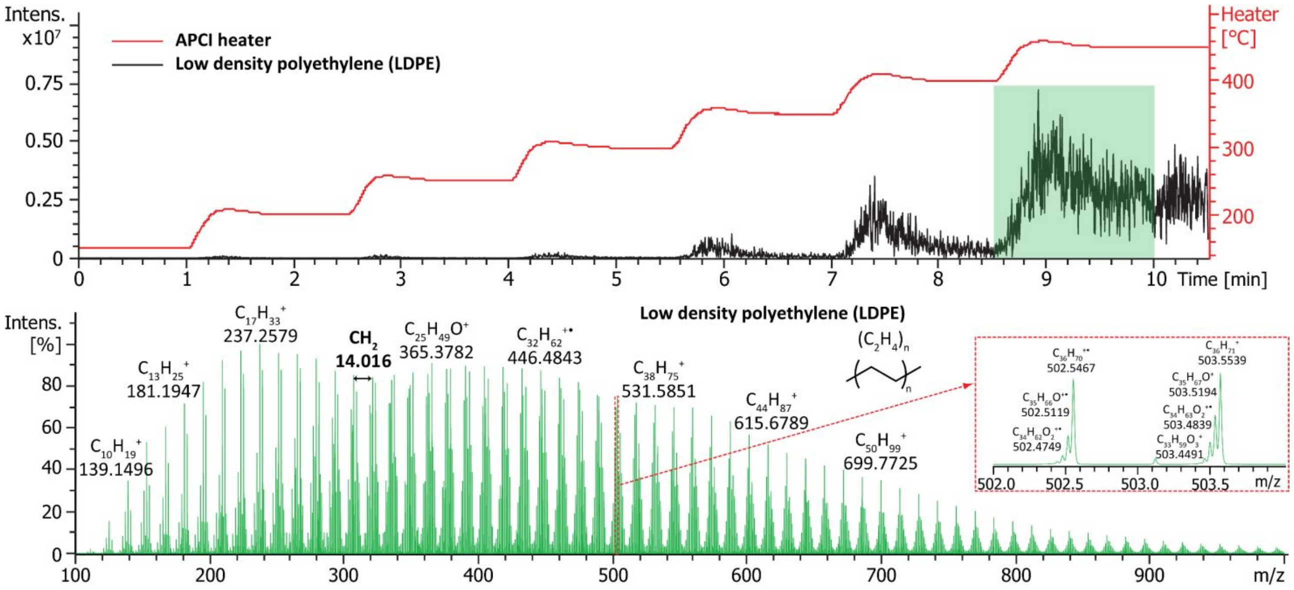

Figure S1. DIP-MS analysis of LDPE. The top panel shows a total ion chromatogram (TIC; black trace) obtained using a vaporizer temperature program from 150 to 450 °C (red trace), whereas the bottom panel presents an averaged mass spectrum obtained at 450 °C.

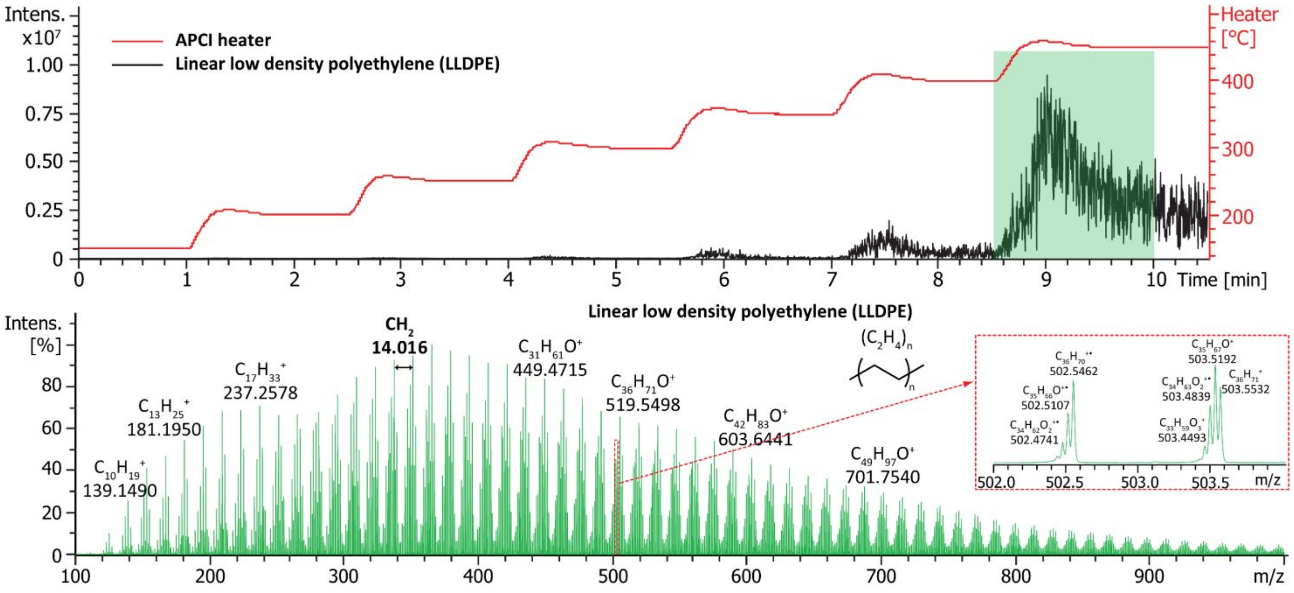

Figure S2. DIP-MS analysis of LLDPE. The top panel shows a TIC (black trace) obtained using a vaporizer temperature program from 150 to 450 °C (red trace), whereas the bottom panel presents an averaged mass spectrum obtained at 450 °C.

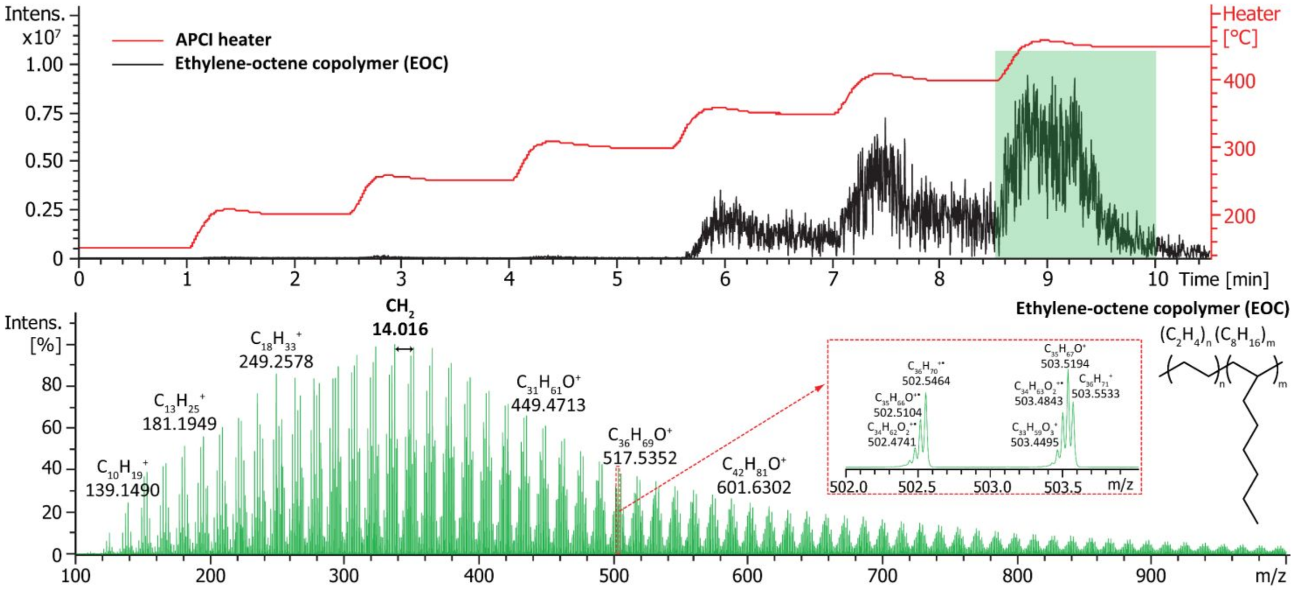

Figure S3. DIP-MS analysis of EOC. The top panel shows a TIC (black trace) obtained using a vaporizer temperature program from 150 to 450 °C (red trace), whereas the bottom panel presents an averaged mass spectrum obtained at 450 °C.

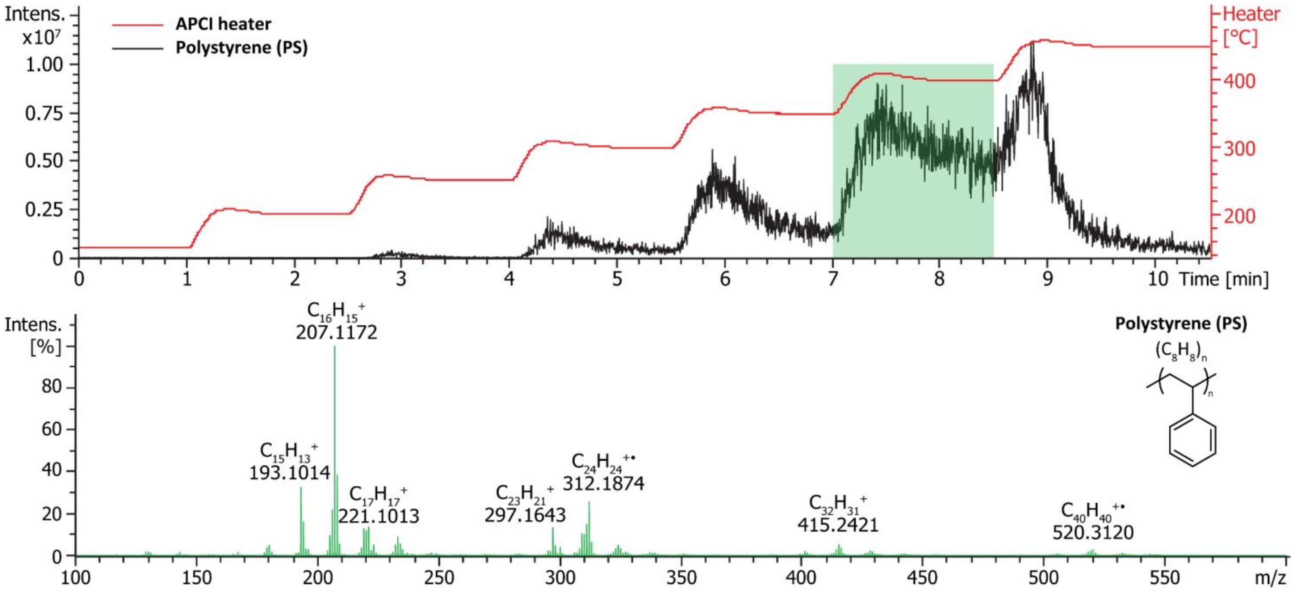

Figure S4. DIP-MS analysis of PS. The top panel shows a TIC (black trace) obtained using a vaporizer temperature program from 150 to 450 °C (red trace), whereas the bottom panel presents an averaged mass spectrum obtained at 400 °C.

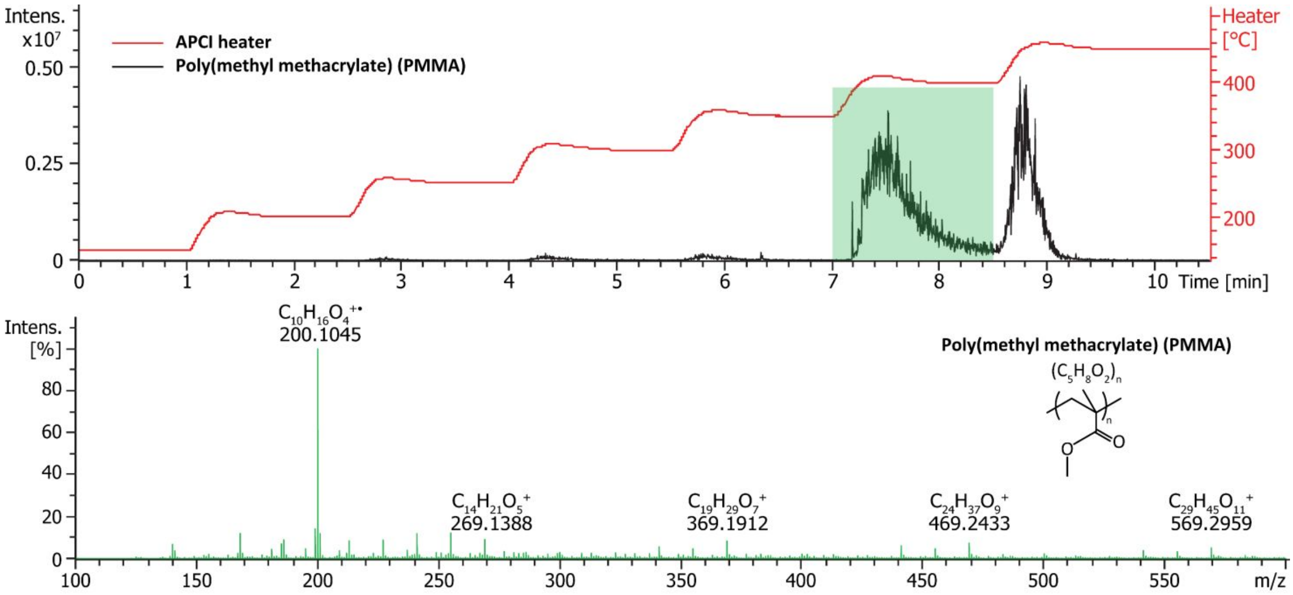

Figure S5. DIP-MS analysis of PMMA. The top panel shows a TIC (black trace) obtained using a vaporizer temperature program from 150 to 450 °C (red trace), whereas the bottom panel presents an averaged mass spectrum obtained at 400 °C.

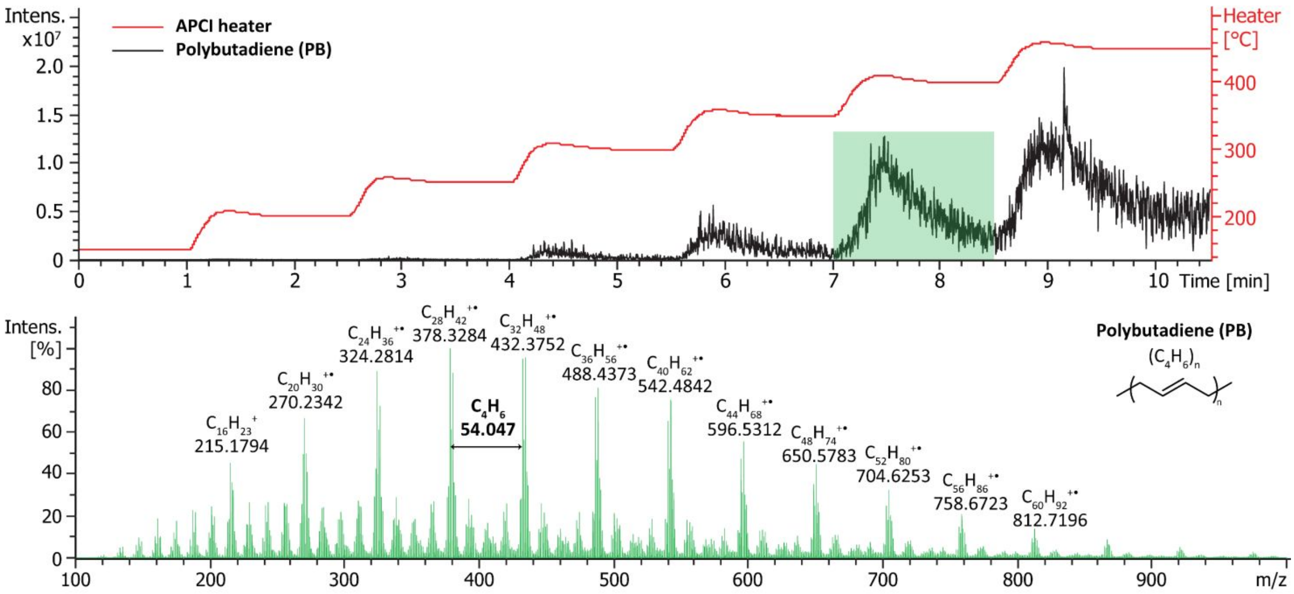

Figure S6. DIP-MS analysis of PB. The top panel shows a TIC (black trace) obtained using a vaporizer temperature program from 150 to 450 °C (red trace), whereas the bottom panel presents an averaged mass spectrum obtained at 400 °C.

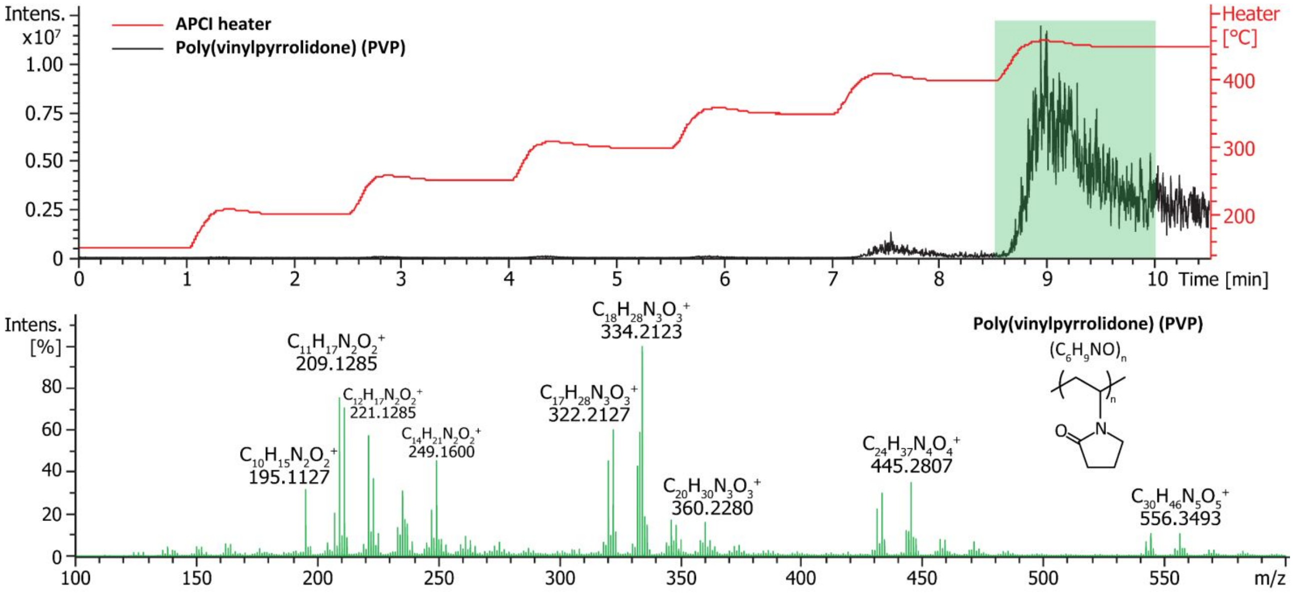

Figure S7. DIP-MS analysis of PVP. The top panel shows a TIC (black trace) obtained using a vaporizer temperature program from 150 to 450 °C (red trace), whereas the bottom panel presents an averaged mass spectrum obtained at 450 °C.

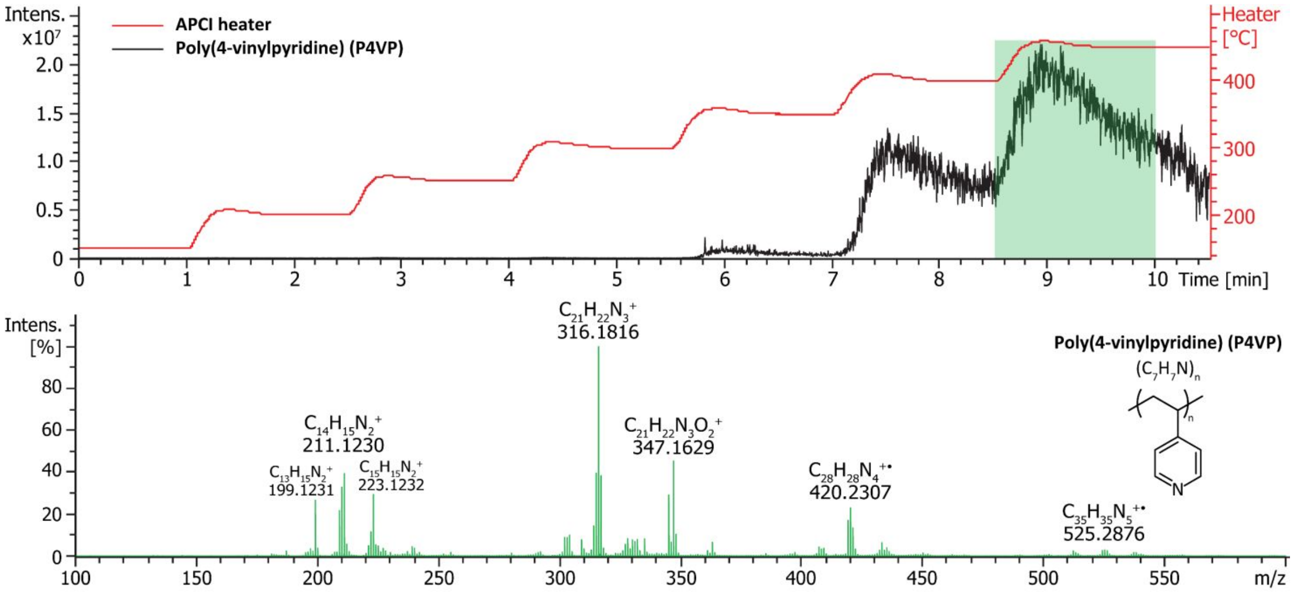

Figure S8. DIP-MS analysis of P4VP. The top panel shows a TIC (black trace) obtained using a vaporizer temperature program from 150 to 450 °C (red trace), whereas the bottom panel presents an averaged mass spectrum obtained at 450 °C.

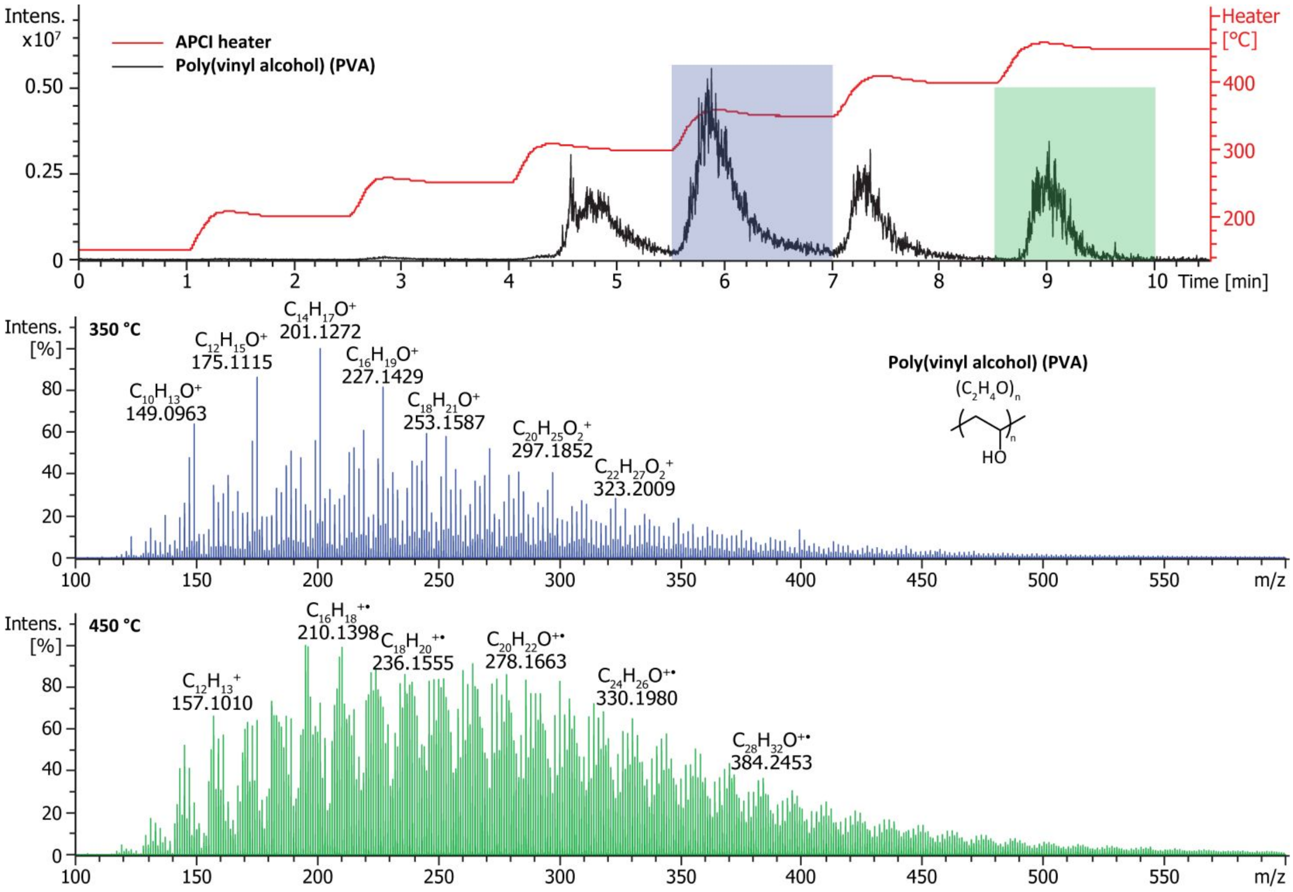

Figure S9. DIP-MS analysis of PVA. The top panel shows a TIC (black trace) obtained using a vaporizer temperature program from 150 to 450 °C (red trace), whereas the middle and bottom panels present averaged mass spectra obtained at 350 and 450 °C, respectively.

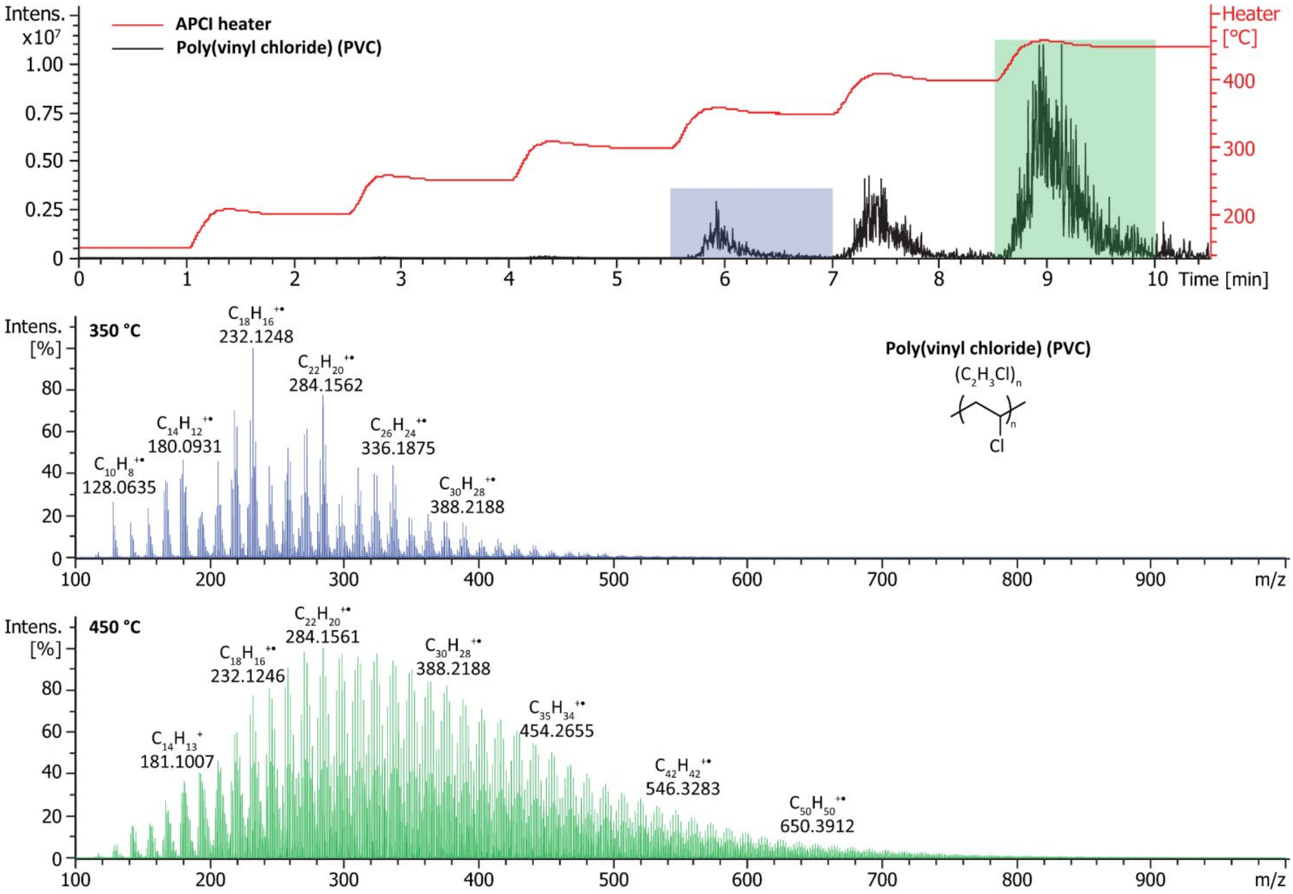

Figure S10. DIP-MS analysis of PVC. The top panel shows a TIC (black trace) obtained using a vaporizer temperature program from 150 to 450 °C (red trace), whereas the middle and bottom panels present averaged mass spectra obtained at 350 and 450 °C, respectively.

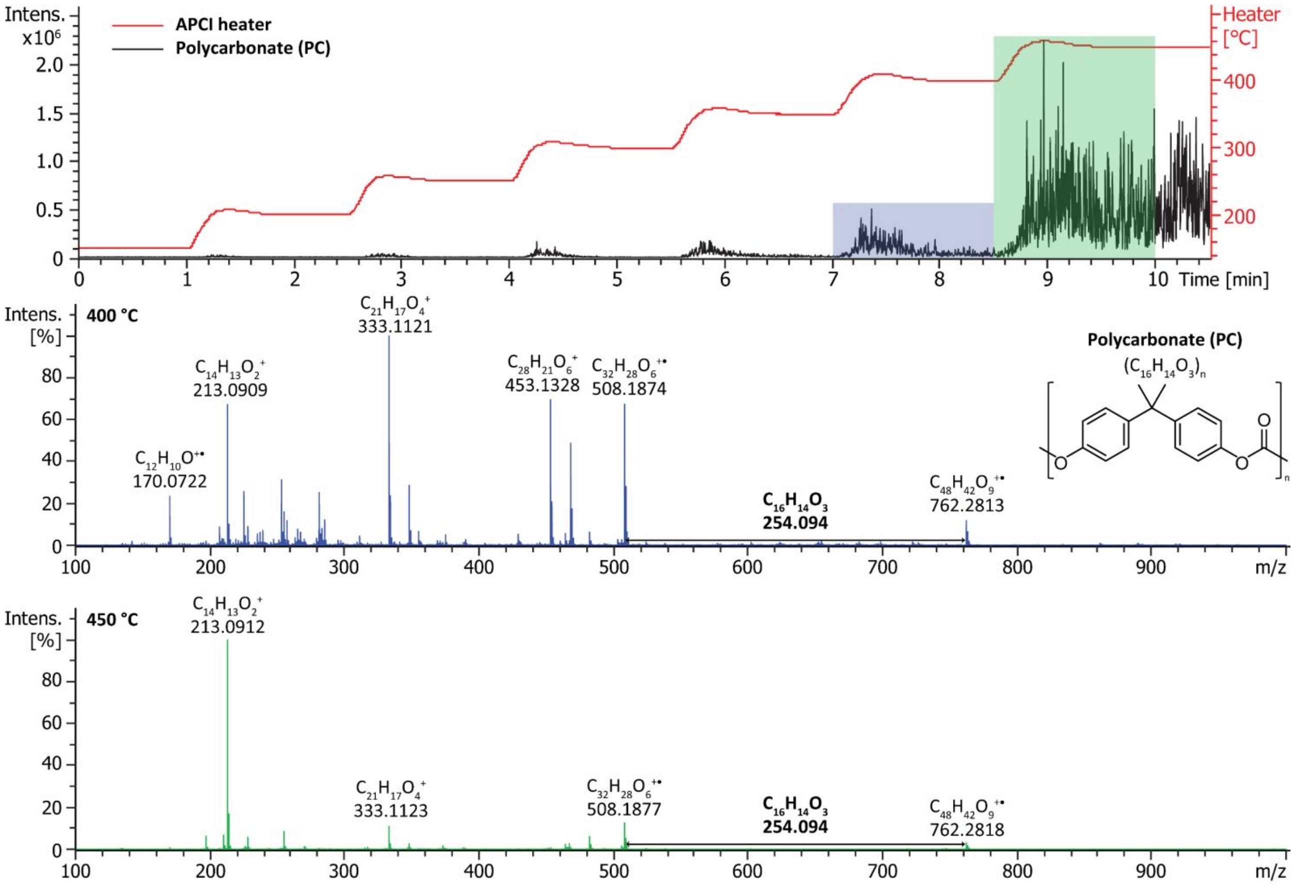

Figure S11. DIP-MS analysis of PC. The top panel shows a TIC (black trace) obtained using a vaporizer temperature program from 150 to 450 °C (red trace), whereas the middle and bottom panels present averaged mass spectra obtained at 400 and 450 °C, respectively.

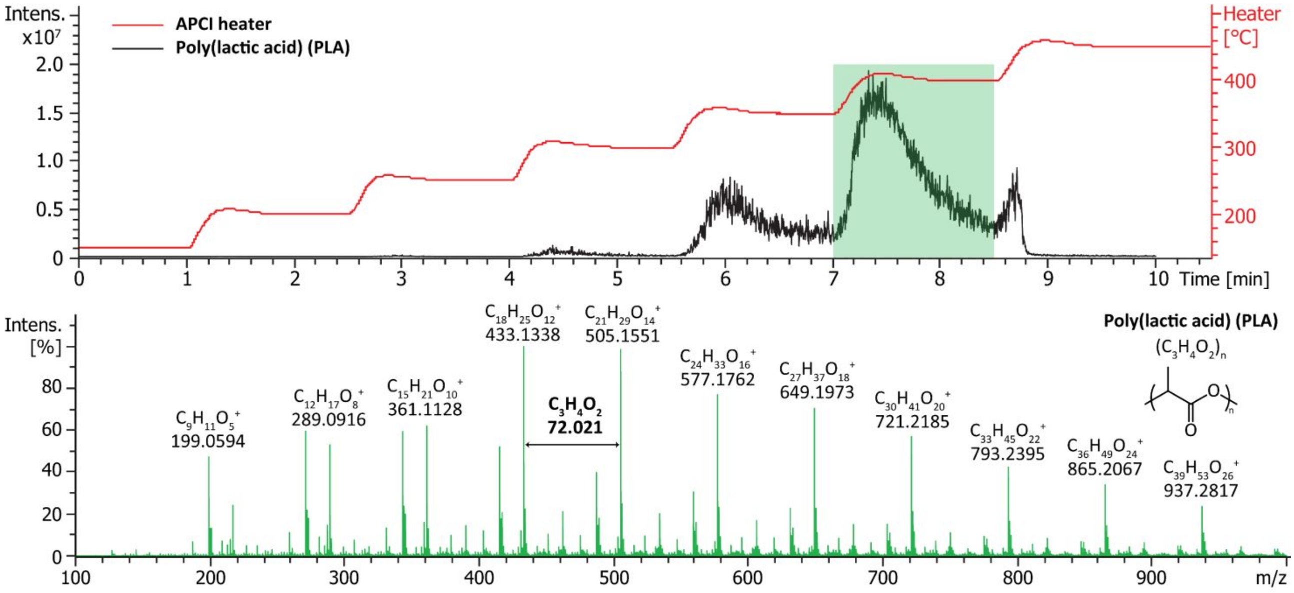

Figure S12. DIP-MS analysis of PLA. The top panel shows a TIC (black trace) obtained using a vaporizer temperature program from 150 to 450 °C (red trace), whereas the bottom panel presents an averaged mass spectrum obtained at 400 °C.

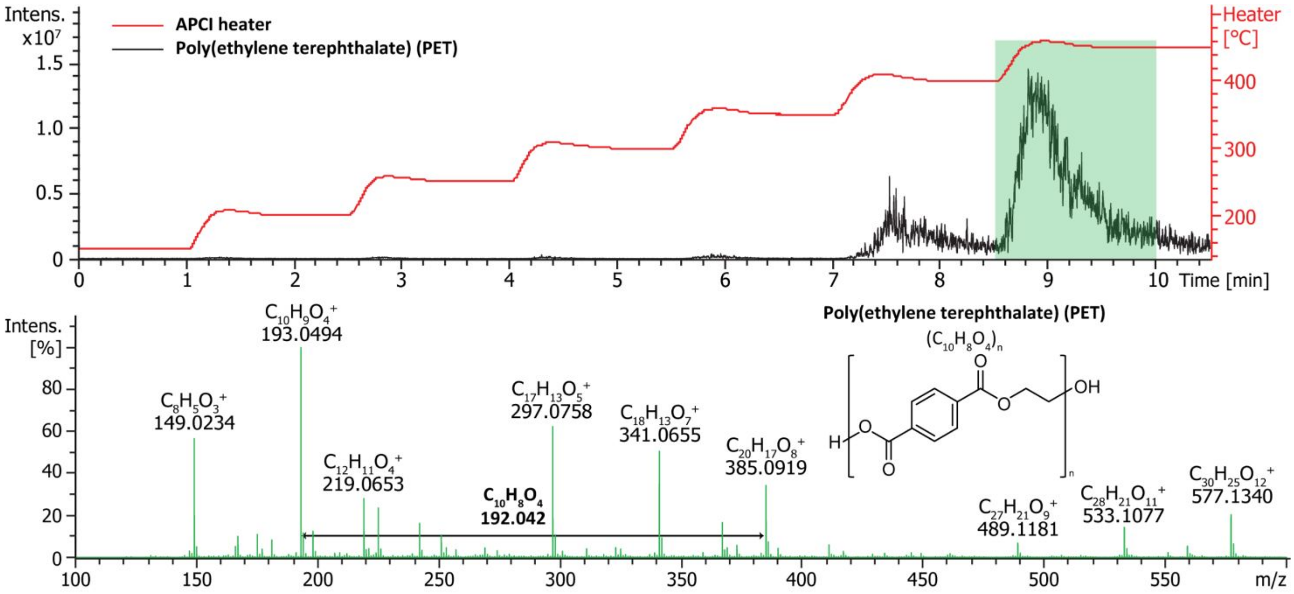

Figure S13. DIP-MS analysis of PET. The top panel shows a TIC (black trace) obtained using a vaporizer temperature program from 150 to 450 °C (red trace), whereas the bottom panel presents an averaged mass spectrum obtained at 450 °C.

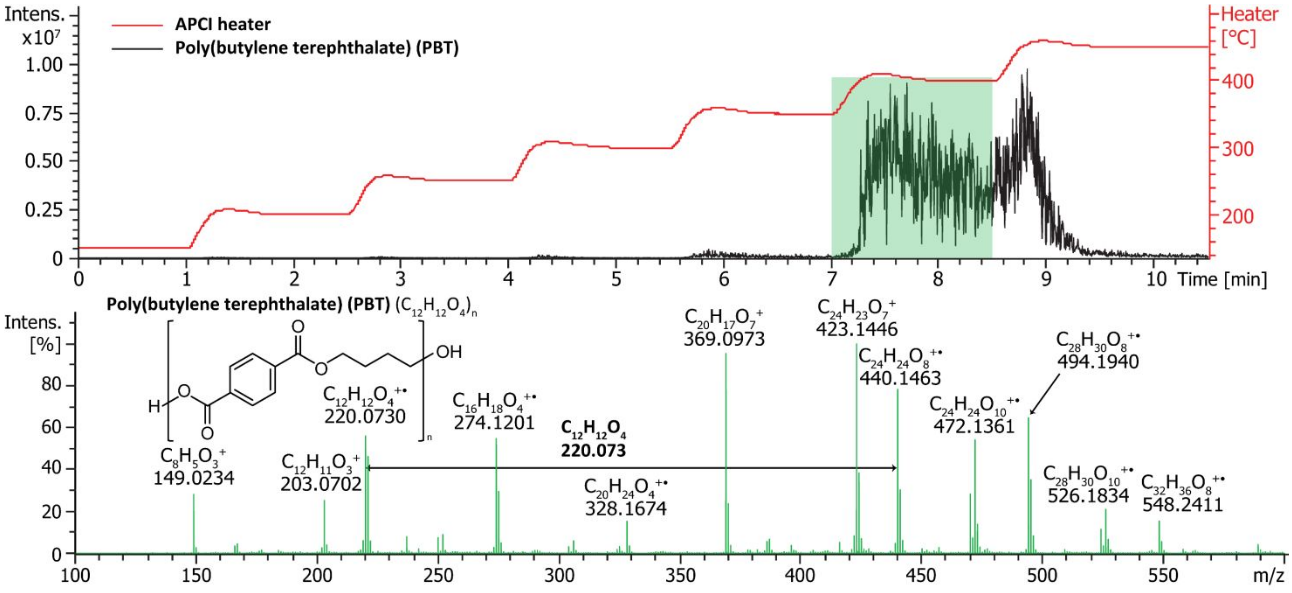

Figure S14. DIP-MS analysis of PBT. The top panel shows a TIC (black trace) obtained using a vaporizer temperature program from 150 to 450 °C (red trace), whereas the bottom panel presents an averaged mass spectrum obtained at 400 °C.

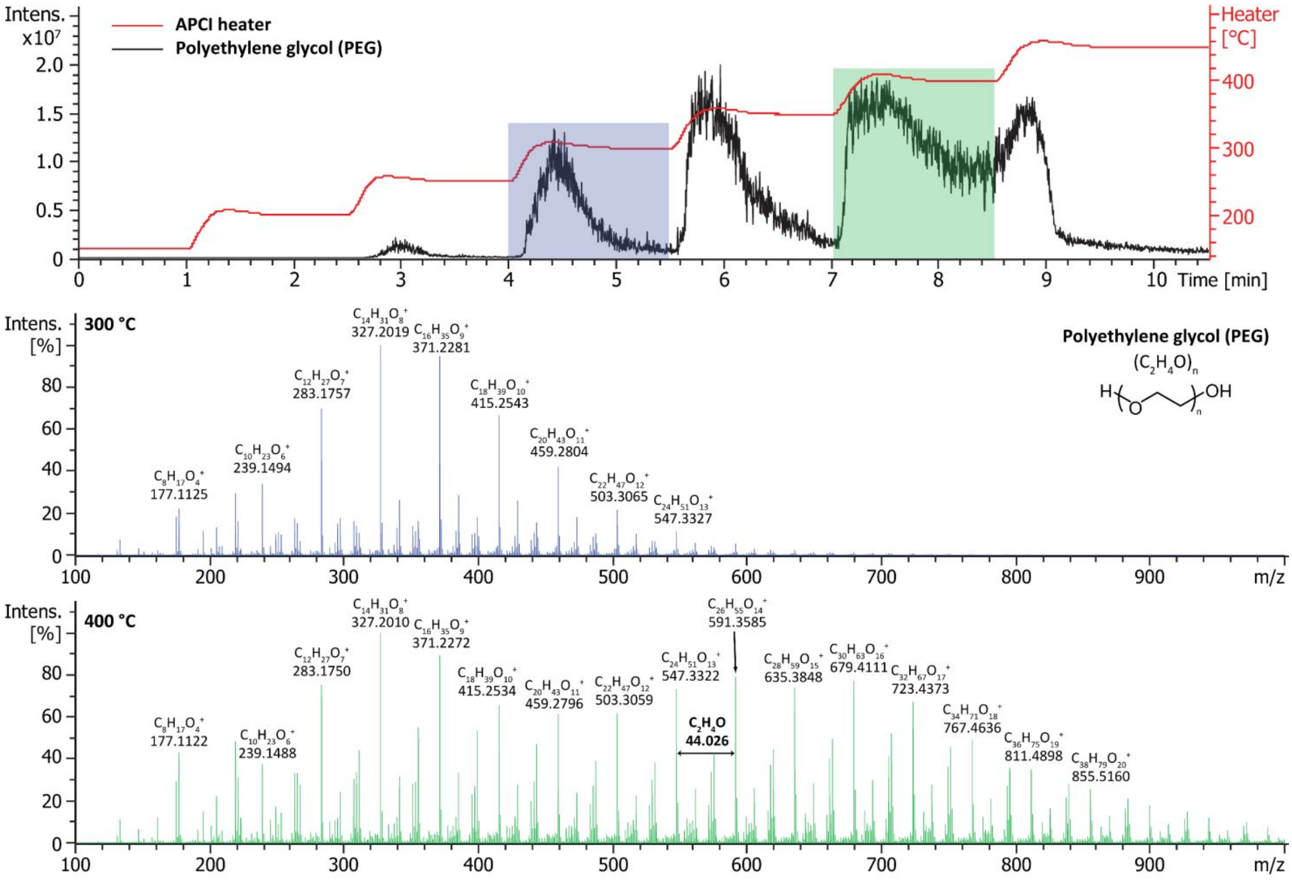

Figure S15. DIP-MS analysis of PEG. The top panel shows a TIC (black trace) obtained using a vaporizer temperature program from 150 to 450 °C (red trace), whereas the middle and bottom panels present averaged mass spectra obtained at 300 and 400 °C, respectively.

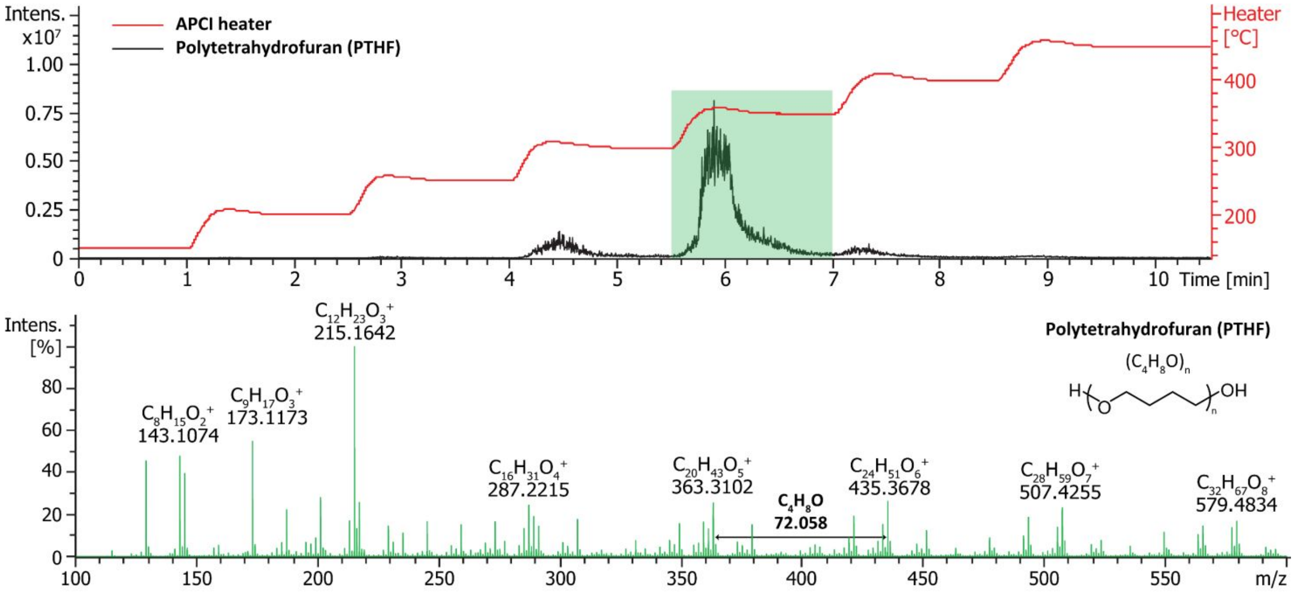

Figure S16. DIP-MS analysis of PTHF. The top panel shows a TIC (black trace) obtained using a vaporizer temperature program from 150 to 450 °C (red trace), whereas the bottom panel presents an averaged mass spectrum obtained at 350 °C.

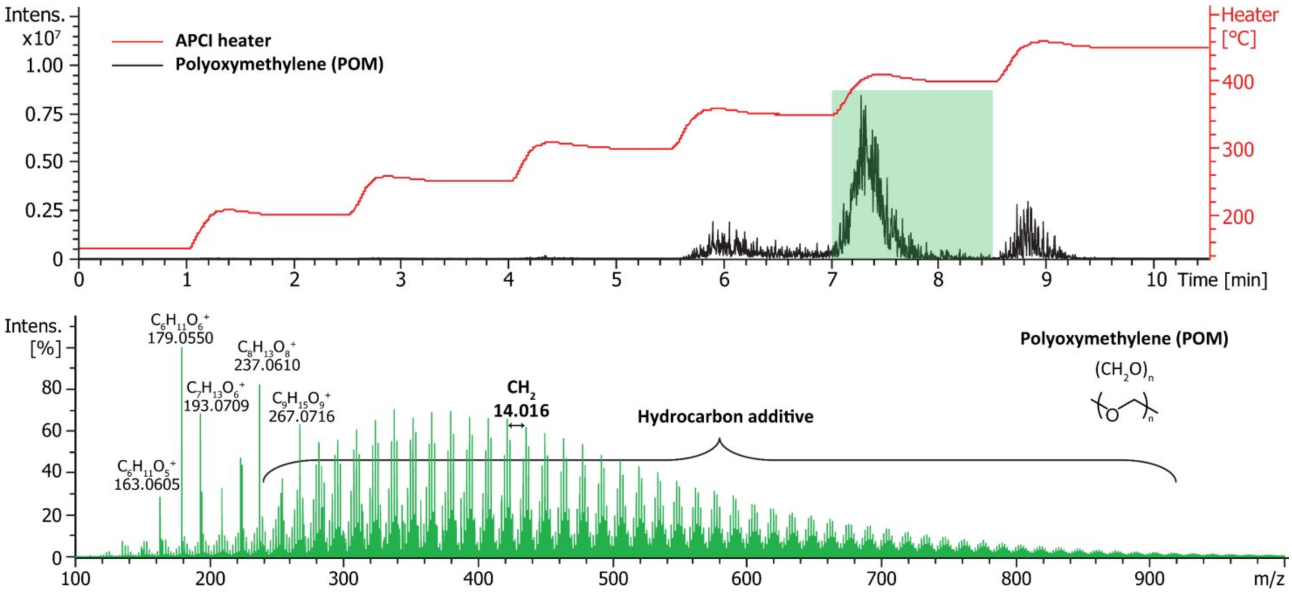

Figure S17. DIP-MS analysis of POM. The top panel shows a TIC (black trace) obtained using a vaporizer temperature program from 150 to 450 °C (red trace), whereas the bottom panel presents an averaged mass spectrum obtained at 400 °C.

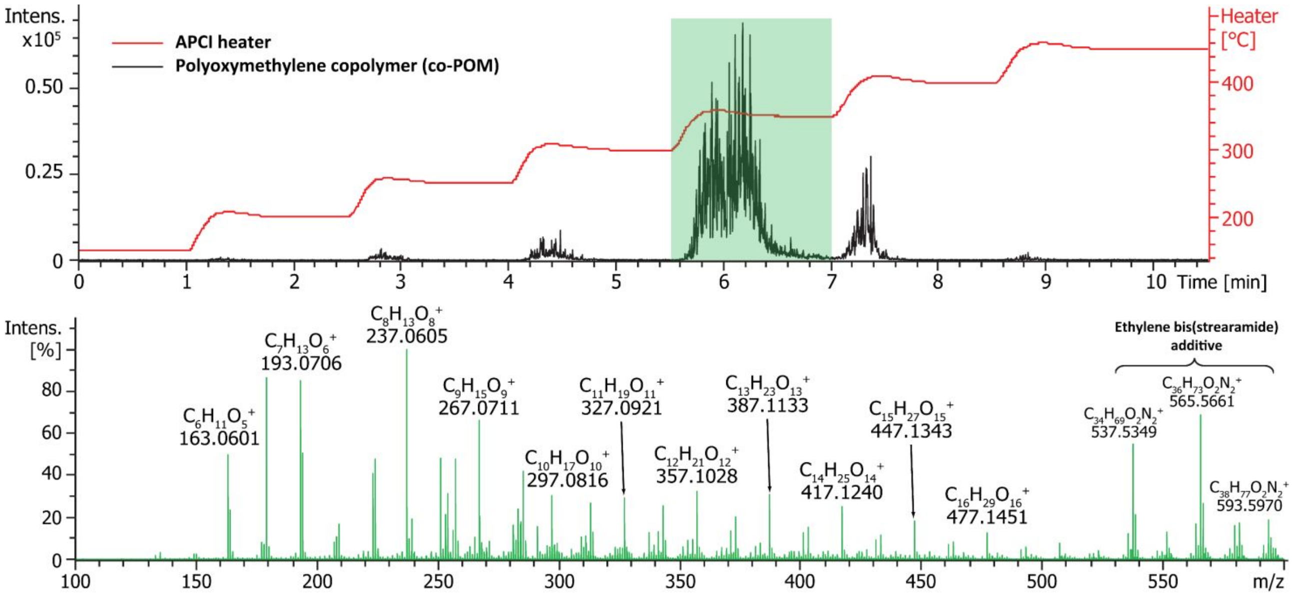

Figure S18. DIP-MS analysis of co-POM. The top panel shows a TIC (black trace) obtained using a vaporizer temperature program from 150 to 450 °C (red trace), whereas the bottom panel presents an averaged mass spectrum obtained at 350 °C.

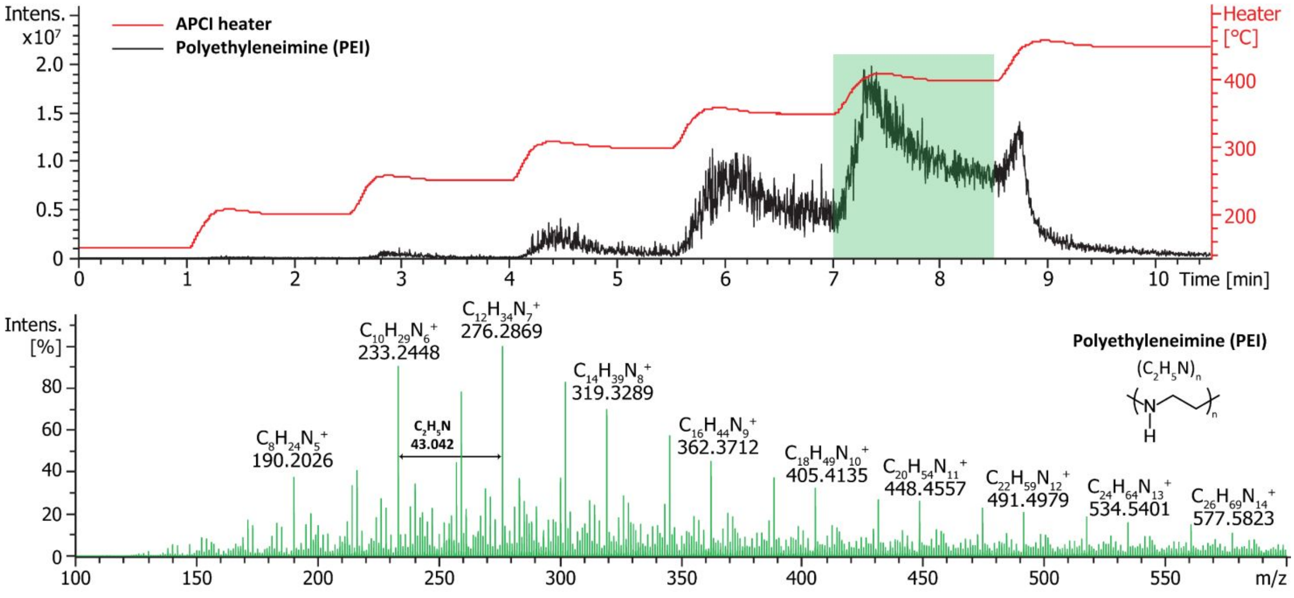

Figure S19. DIP-MS analysis of PEI. The top panel shows a TIC (black trace) obtained using a vaporizer temperature program from 150 to 450 °C (red trace), whereas the bottom panel presents an averaged mass spectrum obtained at 400 °C.

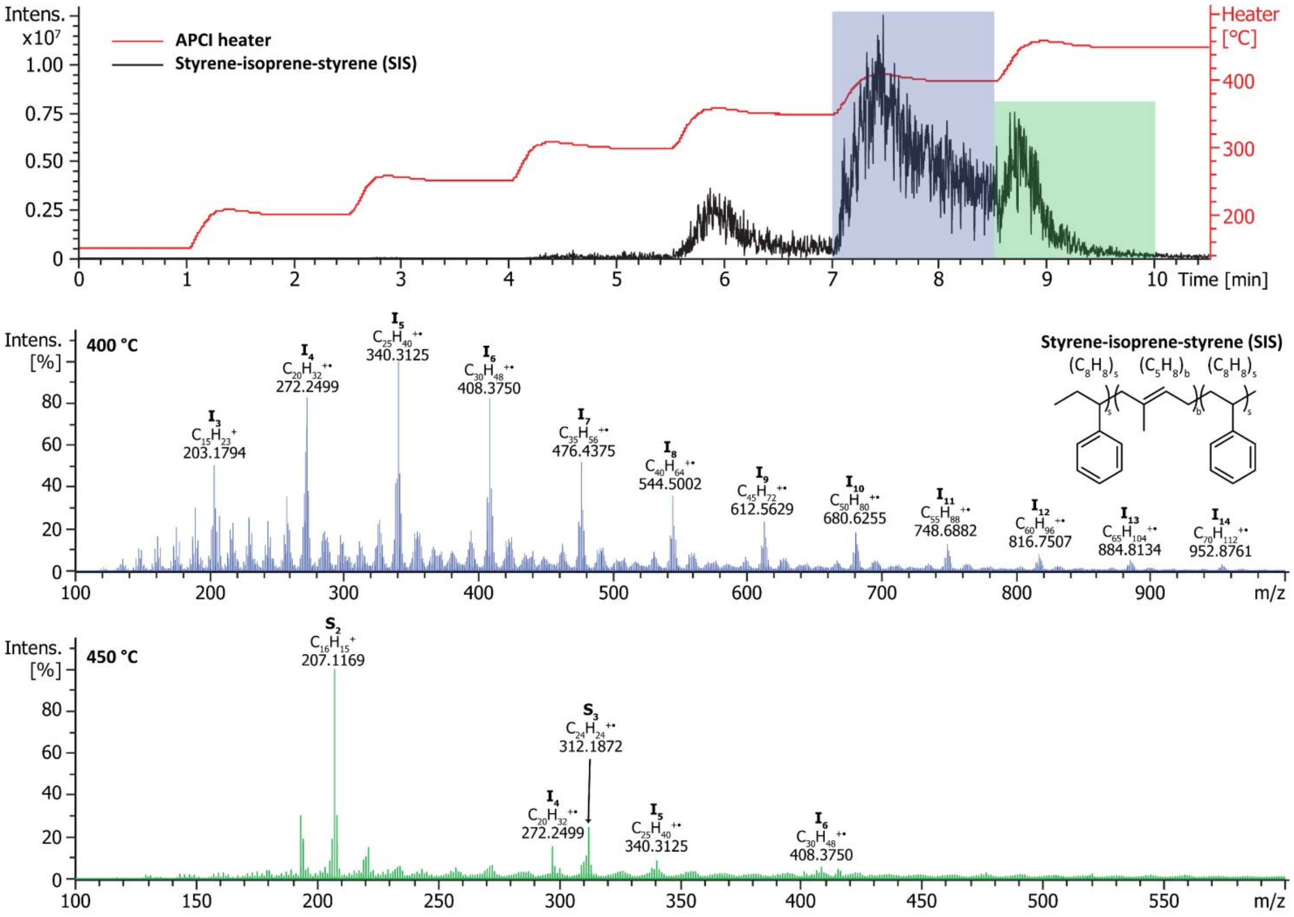

Figure S20. DIP-MS analysis of SIS. The top panel shows a TIC (black trace) obtained using a vaporizer temperature program from 150 to 450 °C (red trace), whereas the middle and bottom panels present averaged mass spectra obtained at 400 and 450 °C, respectively. The observed polymer fragments were designated as S_sI_i, where s and i indicate the number of styrene and isoprene monomer units, respectively.

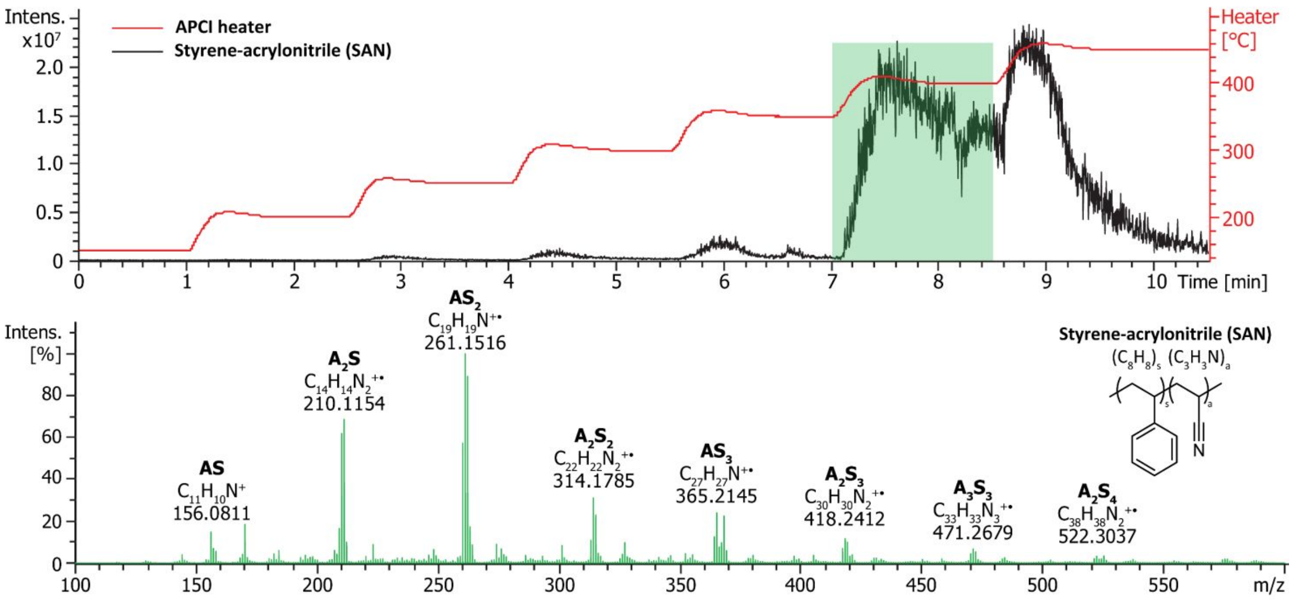

Figure S21. DIP-MS analysis of SAN. The top panel shows a TIC (black trace) obtained using a vaporizer temperature program from 150 to 450 °C (red trace), whereas the bottom panel presents an averaged mass spectrum obtained at 400 °C. The observed polymer fragments were designated as A_aS_s, where a and s indicate the number of acrylonitrile and styrene monomer units, respectively.

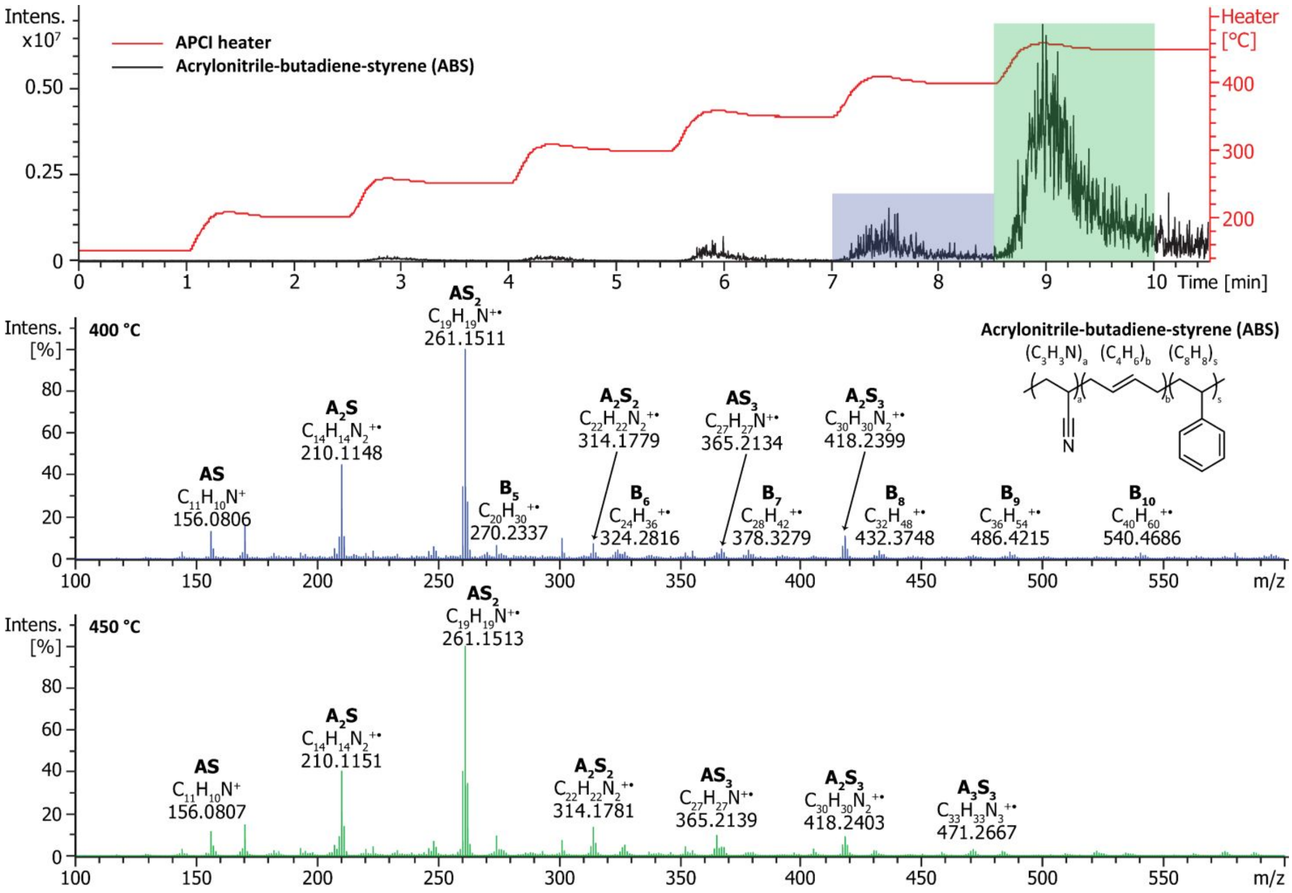

Figure S22. DIP-MS analysis of ABS. The top panel shows a TIC (black trace) obtained using a vaporizer temperature program from 150 to 450 °C (red trace), whereas the middle and bottom panels present averaged mass spectra obtained at 400 and 450 °C, respectively. The observed polymer fragments were designated as $A_aB_bS_s$, where a, b, and s indicate the number of acrylonitrile, butadiene, and styrene monomer units, respectively.

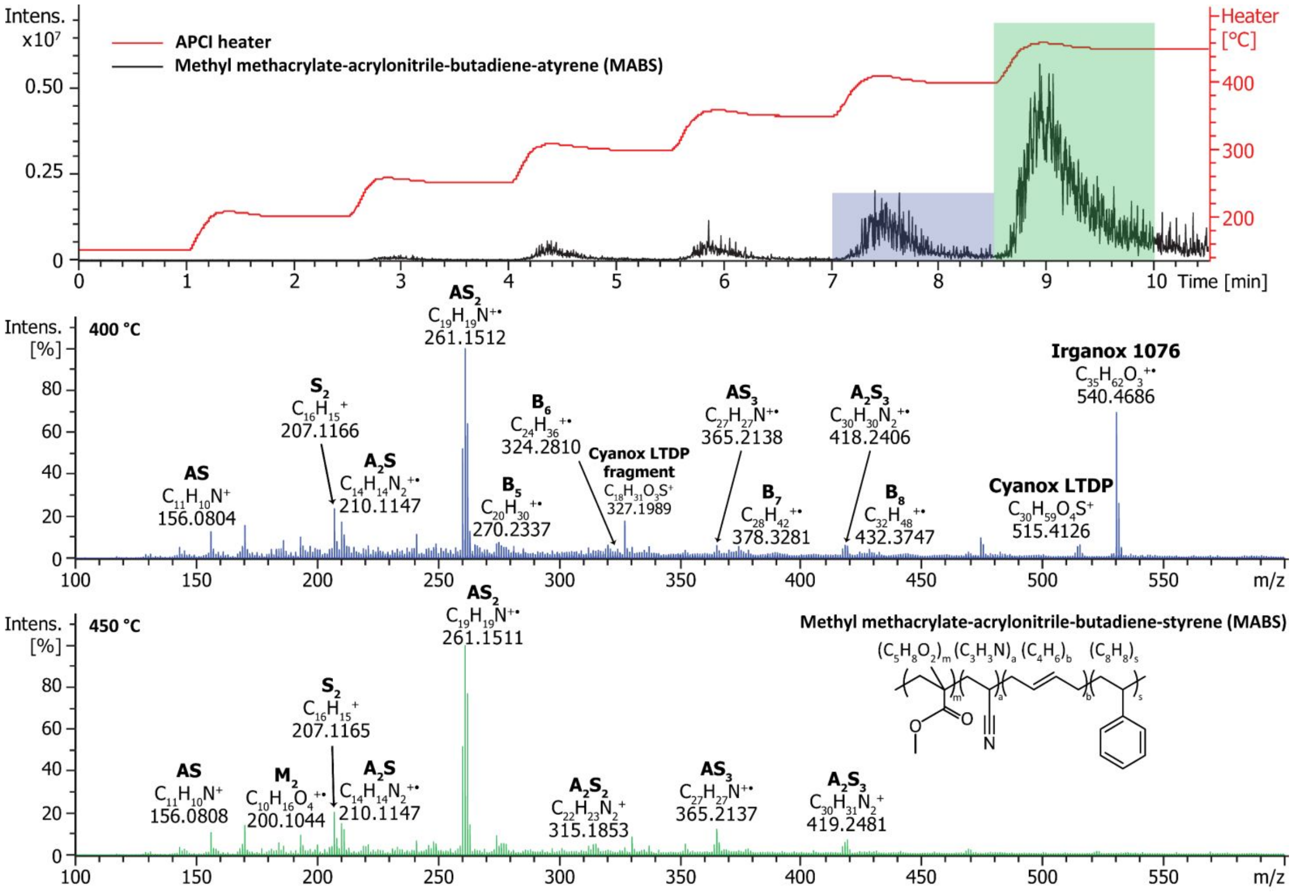

Figure S23. DIP-MS analysis of MABS. The top panel shows a TIC (black trace) obtained using a vaporizer temperature program from 150 to 450 °C (red trace), whereas the middle and bottom panels present averaged mass spectra obtained at 400 and 450 °C, respectively. The observed polymer fragments were designated as $M_mA_aB_bS_s$, where m, a, b, and s indicate the number of methyl methacrylate, acrylonitrile, butadiene, and styrene monomer units, respectively.

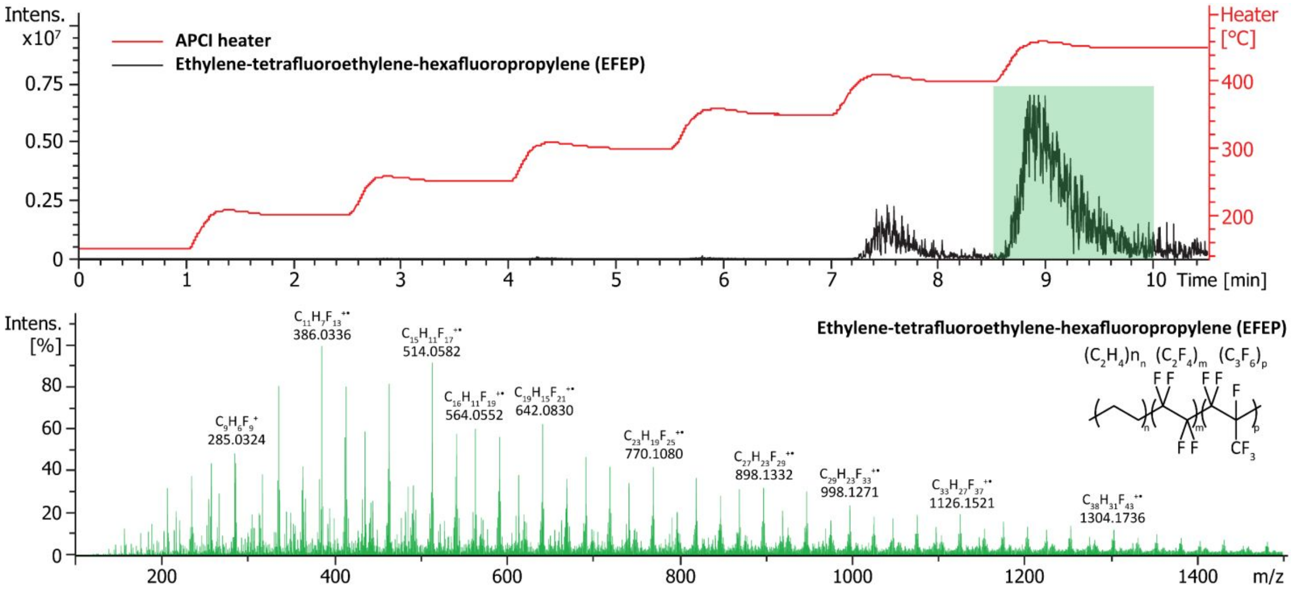

Figure S24. DIP-MS analysis of EFEP. The top panel shows a TIC obtained using a vaporizer temperature program from 150 to 450 °C (red trace), whereas the bottom panel presents an averaged mass spectrum obtained at 450 °C.

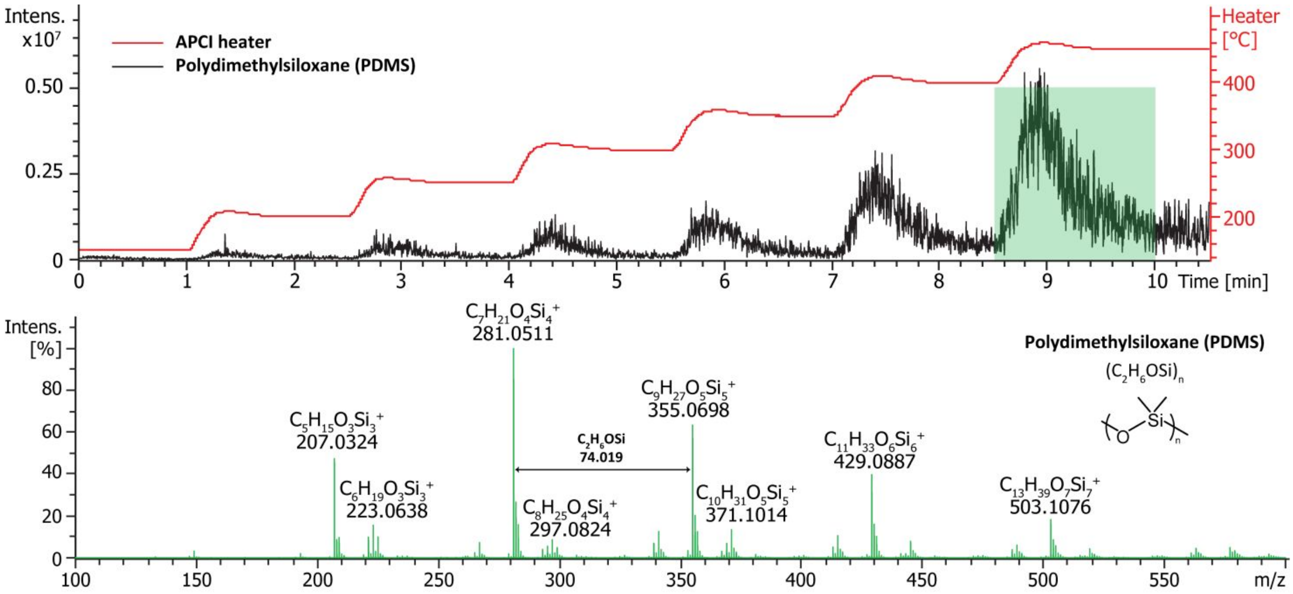

Figure S25. DIP-MS analysis of PDMS. The top panel shows a TIC (black trace) obtained using a vaporizer temperature program from 150 to 450 °C (red trace), whereas the bottom panel presents an averaged mass spectrum obtained at 450 °C.

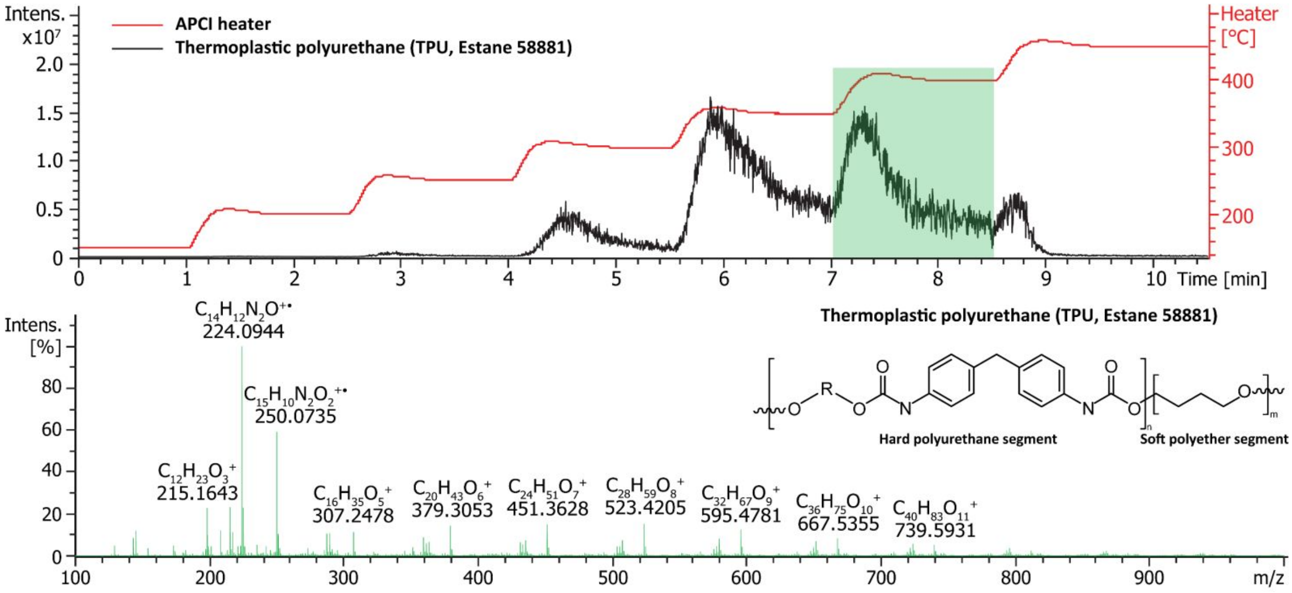

Figure S26. DIP-MS analysis of Estane 58881 polyether type TPU. The top panel shows a TIC obtained using a vaporizer temperature program from 150 to 450 °C (red trace), whereas the bottom panel presents an averaged mass spectrum obtained at 400 °C.

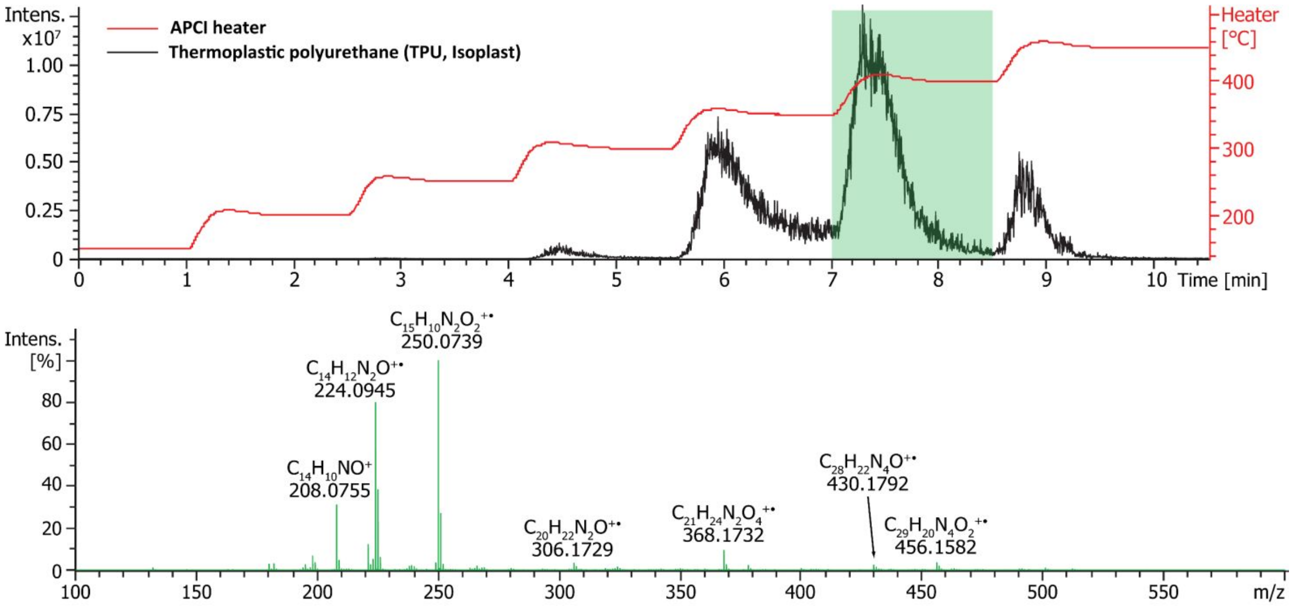

Figure S27. DIP-MS analysis of Isoplast TPU. The top panel shows a TIC obtained using a vaporizer temperature program from 150 to 450 °C (red trace), whereas the bottom panel presents an averaged mass spectrum obtained at 400 °C.

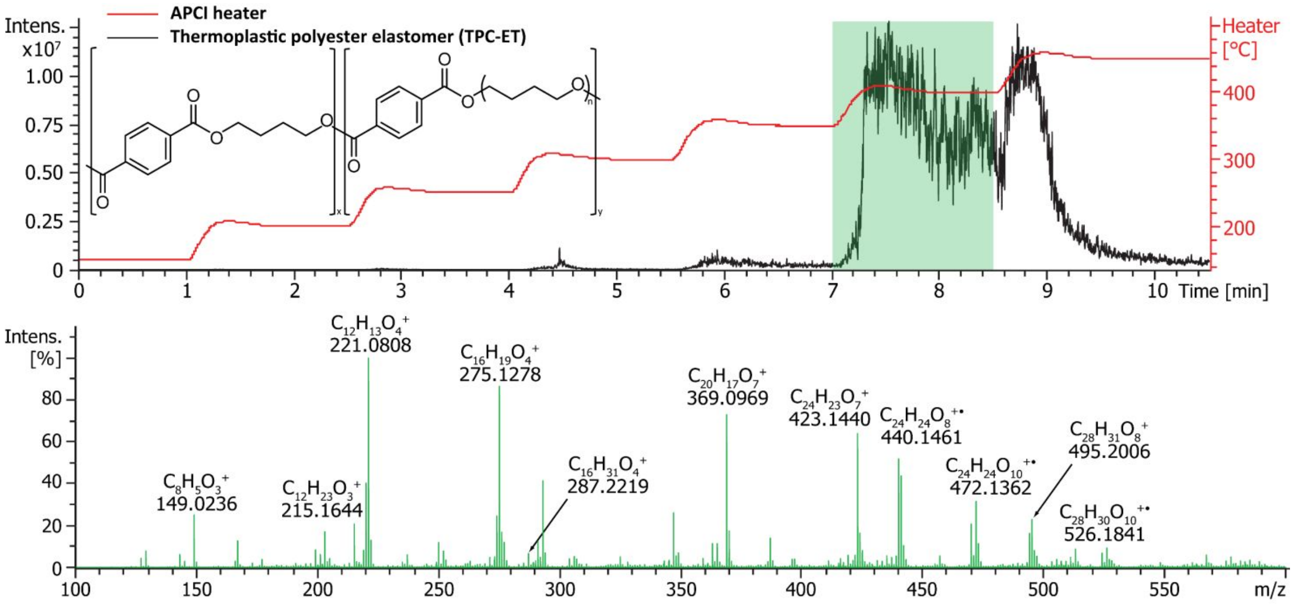

Figure S28. DIP-MS analysis of Hytrel 4056 TPC-ET. The top panel shows a TIC (black trace) obtained using a vaporizer temperature program from 150 to 450 °C (red trace), whereas the bottom panel presents an averaged mass spectrum obtained at 400 °C.
